# Shellmycin A–D, Novel Bioactive Tetrahydroanthra-γ-Pyrone Antibiotics from Marine *Streptomyces* sp. Shell-016

**DOI:** 10.3390/md18010058

**Published:** 2020-01-16

**Authors:** Yong Han, Yan Wang, Yuehan Yang, Haotong Chen

**Affiliations:** 1 School of Basic Medical Science, Shandong University, Jinan 250012, China; 2 Edison Biotechnology Institute, Ohio University, Athens, OH 45701, USA; yy506416@ohio.edu; 3 Collage of marine life science, Ocean University of China, Qingdao 266003, China; wangy12@ouc.edu.cn

**Keywords:** marine *Streptomyces* sp., shell-016, tetrahydroanthra-γ-pyrone, NMR and single crystal X-ray diffraction, stereoisomers, cytotoxic activity

## Abstract

Four novel bioactive tetrahydroanthra-γ-pyrone compounds, shellmycin A–D (**1**–**4**), were isolated from the marine *Streptomyces* sp. shell-016 derived from a shell sediment sample collected from Binzhou Shell Dike Island and Wetland National Nature Reserve, China. The structures of these four compounds were established by interpretation of 1D and 2D NMR and HR-MS data, in which the absolute configuration of **1** was confirmed by single crystal X-ray diffraction, and compound **3** and **4** are a pair of stereoisomers. Compound **1**–**4** exhibited cytotoxic activity against five cancer cell lines with the IC_50_ value from 0.69 μM to 26.3 μM. Based on their structure–activity relationship, the putative biosynthetic pathways of these four compounds were also discussed.

## 1. Introduction

Microbial secondary metabolites with widely divergent chemical structures have been regarded not only as prolific drug candidates, but also an inspiration for the starting point of drug design [1]. For half a century, antibiotics derived from microorganisms have become the leading drugs in different clinical fields due to their distinct structures and excellent activities [2,3]. However, the emerging of drug-resistant bacteria and drug-resistant tumors has prompted people to search for new drugs and novel mechanisms of action [4,5]. More recently, the marine environment is considered as an abundant microbial resource due to its complex ecological environment and relatively harsh living conditions [6,7], which implies there are enormous unexplored secondary metabolites and their biosynthetic gene clusters to be discovered and characterized. Thus, marine microorganisms appear to be a treasure trove of new active microbial drugs [8,9]. Recent studies have fully demonstrated that marine actinomycetes can produce a variety of secondary metabolites with diverse biological activities, including antibacterial [10,11], anti-tumor [12], anti-inflammatory [13], immune regulation [14], anti-parasitic [15], anti-oxidation [16], and anti-virus [17].

The naturally occurring aromatic γ-pyrone structure, a major class of polyketides, is widely found in bioactive natural products, and its structural diversity and various biological activities make it important in the pharmaceutical industry [18,19]. For example, the flavonoids widely distribute in plant are benzopyrone derivatives [20]. The (bis-) naphtho-γ-pyrones compounds are mostly discovered in secondary metabolites of fungi [21]. Some compounds with more complex structures, which contain anthraquinone-γ-pyrone structural units, such as parimycin [22], saliniquinones [23], and hedamycin [24] are present in secondary metabolites of bacteria. Given the significant drug development potential of this class of compounds, it is of great interest to discover new compounds containing anthraquinone-γ-pyrone core in bacterial secondary metabolites [25].

**Figure 1:**
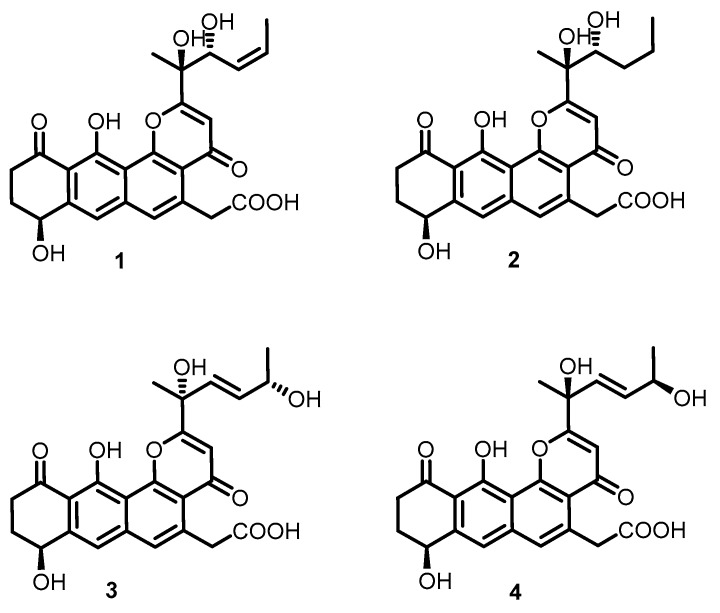
Structures of compounds **1**–**4**.

Binzhou shell island is one of the three old shell ridges in the world which formed over thousands of years in the Yellow River Delta, representing a great repertoire for the study of the marine species biodiversity [26,27]. Its unique geographical environment results in a unique ecosystem, which raises unique microorganisms producing novel secondary metabolites [28]. In this study, the chemical composition of the marine *Streptomyces* sp. shell-016 from the Binzhou shell dike island was studied. Four new polyketides, named shellmycin A–D (**1**–**4**), containing the tetrahydroanthra-γ-pyrone skeleton, were isolated (Figure 1). The structures and relative configurations of the compounds were elucidated by NMR and high-resolution mass spectrometry (HR-MS). The absolute configuration of **1** was clarified by X-ray single crystal diffraction. This study also investigated the antibacterial activity and anticancer activity of **1**–**4**, indicating that this group of compounds has moderate antibacterial activity and strong anticancer activity.

## 2. Results and Discussion

### 2.1. Extraction and Purification

The strain *Streptomyces* sp. shell-016 was cultured for 11 days on ISP3 agar plates at 28 °C. The 10 L fermented agar were diced and extracted overnight three times with ethyl acetate: methanol: acetic acid (80:15:5, v/v) at room temperature. After removing the organic solvents under vacuum, the crude extract was partitioned between double-distilled water and ethyl acetate (1:1, v/v). Then the ethyl acetate extract was dried with sodium sulfate (anhydrous) and partitioned between methanol and petroleum ether (1:1, v/v). The methanol layer was concentrated under vacuum to obtain the defatted methanol extract (3.9 g), which was subjected to MPLC over reversed phase C-18 silica gel, Sephadex LH-20 and HPLC to yield compounds **1**–**4** (Figure 1).

### 2.2. Structure Elucidation

**Figure 2:**
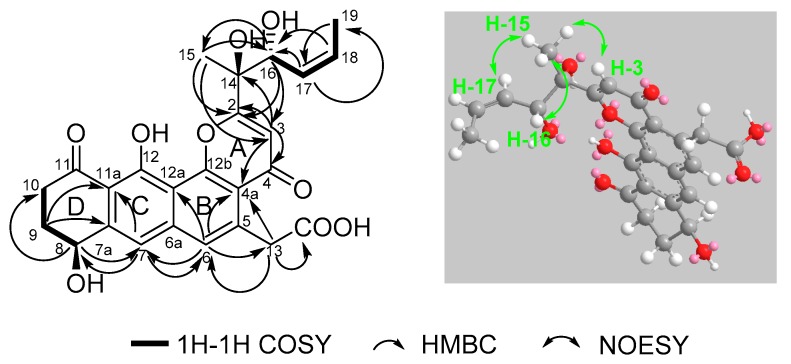
Selected ^1^H–^1^H COSY, HMBC and NOESY correlations for shellmycin A (**1**).

**Figure 3:**
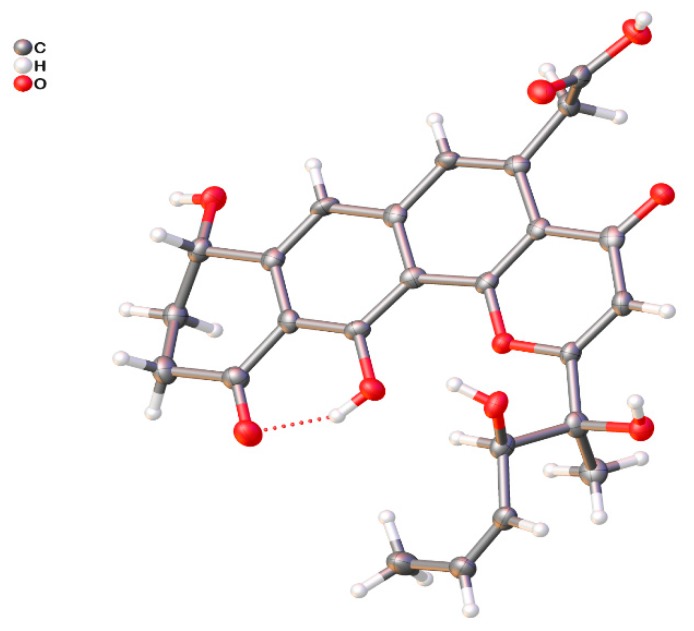
ORTEP drawing of shellmycin A (**1**).

Compound **1** was isolated as a yellow oil, and a yellow crystal was obtained by recrystallization with methanol/acetone (5:1) mixed solvent. The high resolution ESI-MS gave a quasi-molecular ion at *m*/*z* 469.1493 [M + H]^+^ (Figure S1), which is consistent with the molecular formula C_25_H_24_O_9_ (calcd. for C_25_H_25_O_9_^+^, 469.1493). The NMR spectra of **1** (Figures S2–S7 and Table 1) displayed 12 signals, including two methyls, three methylenes, three olefinic protons, and two aromatic protons. The ^13^C-NMR spectroscopic data of **1** showed three carbonyl signals at δ_C_ 175.7, 182.1, and 185.4, and eight quaternary carbon signals (δ_C_ 165.8, 159.1, 147.8, 142.6, 138.0, 120.6, 114.7, and 113.3), which are characteristics for the existence of the ring-fused aromatic system. The presence of ^1^H-^1^H spin–spin coupling system in the COSY experiment of **1** from H-8 to H-9 and H-9 to H-10 combined with the HMBC correlations from H-10 to C-11 and C-11a, and another HMBC correlations from H-8 to C-7a and C-7 confirmed the structure of ring A. The chemical shift of H-8 was as high as 4.95 ppm indicated that it was connected with a hydroxyl group. Two aromatic proton signals suggested that this compound has a multi-substituted naphthalene structure. Additionally, the HMBC correlations from H-7 to C-8, C-6 and C-11a combined with the HMBC correlations from H-6 to C-7, C-13, C12a, C-4a revealed the substitution of naphthalene rings. More in-depth, the chemical shift of C-12 moved to the downfield at 165.8 ppm, suggesting that the C-12 was connected with an oxygen atom. Then, the structure of ring B, C, and D was confirmed. The distinct signals of H-13 (δ_H_ 4.16, 4.26) and the HMBC correlations from this nucleus to C-13-COOH, C-6, and C-4a gave the information that the carboxymethyl group was connected with C-5 on the naphthalene core. The γ-pyrone structure (ring A) formed by C-2, C-3, C-4, C-4a, and C-12b was also deduced by the HMBC correlations from H-3 to C-2, C-4, C-4a. According to the ^1^H-^1^H COSY of compound **1**, there was still a very significant spin-spin coupling system from H-19 to H-18 then to H17 and stopped at H-16. The chemical shift of H-17 (5.73 ppm) and H-18 (5.88 ppm) suggested an ethylenic bond between C-17 and C-18. It was clear to verify the geometry of the C-17/18 double bond is *Z* according to the *cis*-^1^H–^1^H coupling between H-17 and H-18 (*J* = 11.7 Hz) and the single crystal X-ray diffraction. The side chain structure was elucidated by HMBC correlations from H-17 to C-16, H-16 to C-2, C-3, and C-15 combined with the HMBC correlations from H-15 to C-2, C-3, C-16, and from H-3 to C-14. Then the planar structure of compound **1** was illustrated. As shown in Figure 2, the NOESY cross-peaks from H-3 to H-15, H-15 to H-16, and H-15 to H-17 (Figure 2, mixing time 700 ms) were observed but that cannot give definite evidence to support the 14S*, 16R* relative configuration. Compound **1** can form fine crystals in methanol and acetone mixtures, so the single crystal X-ray diffraction was tested, and the absolute configuration of compound **1** was determined (Figure 3, Crystal structure reports see Supplementary Materials; CCDC deposition number: 1970563). Based on the above analyses, the gross structure of **1** was deduced as a new polyketide, for which we proposed the name shellmycin A.

**Figure 4:**
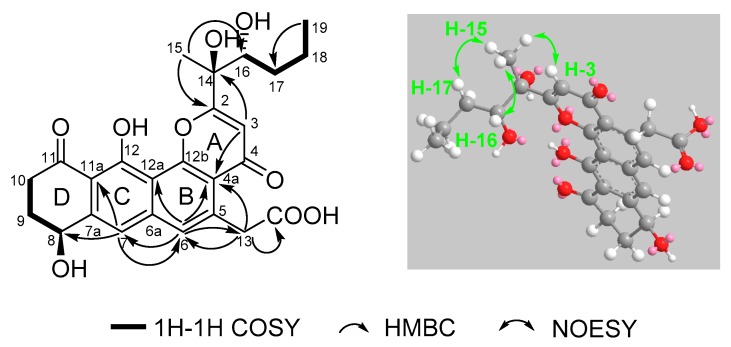
Selected ^1^H–^1^H COSY, HMBC, and NOESY correlations for shellmycin B (**2**).

**Figure 5:**
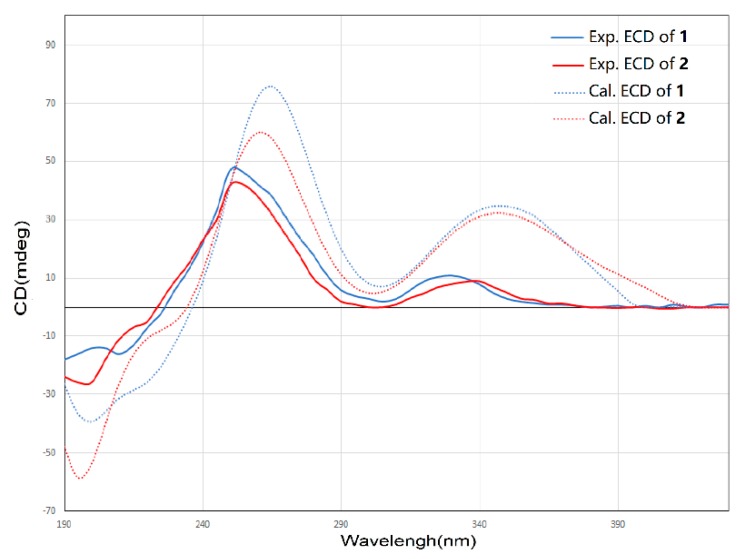
Comparison of the experimental ECD spectra of **1** and **2**.

Compound **2** was isolated as a yellow oil. The molecular formula of **2** was assigned as C_25_H_26_O_9_ by interpretation of a protonated molecular ion peak at *m*/*z* 471.1648 [M + H]^+^ (calcd. for C_25_H_27_O_9_^+^, 471.1650) in the HR-MS spectrum (Figure S8). Compared to **1**, compound **2** has two more hydrogen atoms. The NMR data (Figures S9–S14) demonstrated that compound 2 has a multi-substituted naphthalene structure (Table 2), just like **1**. The same noticeable ^1^H-^1^H COSY cross-peaks from H-10 to H-9 and H-9 to H-8 additional with the HMBC correlation from H-10 to C-11a and H-8 to C-7 suggested the structure of the ring A is the same as compound **1**. Similarly, the HMBC correlation from H-7 to C-8, C-6, and another HMBC correlation from H-6 to C-7, C-13, and C-4a as well as the HMBC correlation from H-13 to C-6 and C-4a suggested that the compound **2** has the same naphthalene core with **1** (Figure 4). Similarly, the HMBC correlation from H-3 to C-4a, C-14, and C-2 could also ascertain the γ-pyrone structure, and its substitution type was the same as **1**. The apparent difference of the ^1^H-NMR and ^13^C-NMR between **2** and **1** was that the characteristic peaks of the double bond disappeared. Distinct ^1^H-^1^H COSY cross peaks from H-16 to H-17 then from H-17 to H-18 then to H-19, and their HMBC correlations gave the evidence, which confirmed the side chain structure of compound **2**. Same as **1**, the NOESY cross-peaks from H-3 to H-15, H-15 to H-16, and H-15 to H-17 cannot give definite evidence to support the 14S*, 16R* relative configuration. The 8S* relative configuration was confirmed from the single-crystal structure of 1 and proposed biosynthetic pathways. Based on comparison of the ECD spectra between **1** and **2**, the absolute configuration of **2** was assigned (Figure 5). Thus, the structure of shellmycin B (**2**) was proposed. 

**Figure 6:**
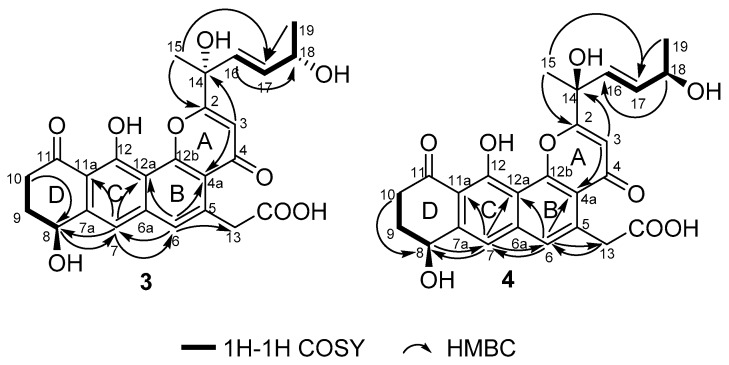
Selected ^1^H–^1^H COSY and HMBC correlations for shellmycin C (**3**) and shellmycin D (**4**).

**Figure 7:**
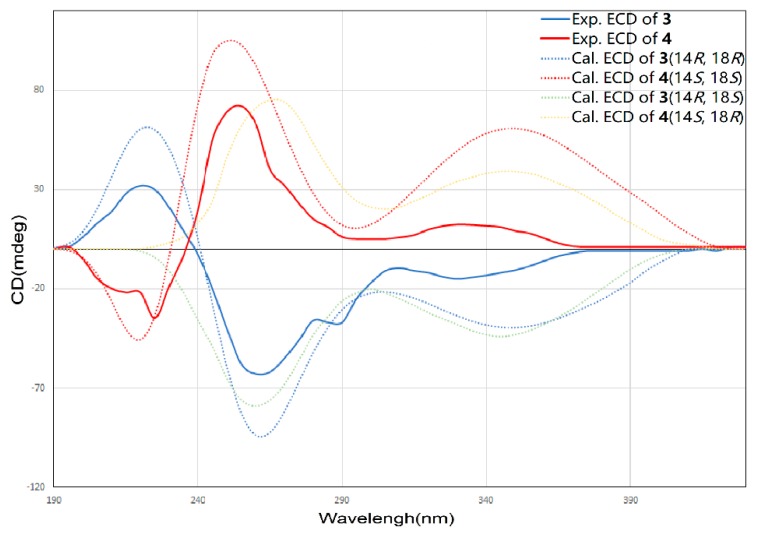
Comparison of the experimental and calculated ECD spectra of **3** and **4**.

The molecular formula of **3** and **4** were both assigned as C_25_H_26_O_9_ by interpretation of a protonated molecular ion peak at *m*/*z* 469.1491 and 469.1494 (calcd for C_25_H_25_O_9_^+^, 469.1493) in the HR-MS spectrum (Figures S15 and S22). Interestingly, these two compounds had almost identical NMR spectra. These facts showed that compound **1** and **4** have the same planar structure. The comparison of its ^1^H and ^13^C NMR data (Table 3 and Table 4, Figures S16–S21; Figures S23–S28) with those of **1** indicated that both structures contain the same backbone fused with the γ-pyrone and naphthalene ring. The ^1^H-^1^H COSY from H-19 to H-18 (δ_H_ 4.32) gave the evidence that C-18 is connected with a hydroxy-substituted carbon (C18, δ_C_ 69.4). Then the four-carbon chain from C-16 to C-19 was established according to ^1^H–^1^H COSY correlations, along with the HMBC correlations from the protons of H-16, H-17, H-18, and H-19 to the corresponding carbons (Figure 6). The geometry of the C-16/17 double bond was determined to be *E*-form based on the *trans*-^1^H–^1^H coupling constants between H-16 and H-17 (*J* = 15.5 Hz). The 8S* relative configuration was proposed based on analogy to the single-crystal structure of **1** and proposed biosynthetic pathways. Most impressive of all, the **3** and **4** have the same planar structure but difference retention time between **3** and **4** is likely due to the stereochemistry of C-14 and C-18. In compound **4**, a distinct NOESY cross peak was found between H-15 and H-16, but there was not any signal between H-15 and H-17. A very different NOESY correlation from H-15 to both H-16 and H-17 suggested that there have some difference between **3** and **4**. Based on the ECD spectra and calculated spectra of **3** and **4**, the absolute configurations of **3** and **4** were assigned (Figure 7) that the stereochemistry of C-14 should be R* in **4** but S* in **3**. Thus, **3** and **4** are a pair of stereoisomers, called shellmycin C (**3**) and shellmycin D (**4**).

### 2.3. Bioactivity Assay

The MICs were determined to evaluate the antimicrobial activities of the shellmycins. *Candida albicans* ATCC18804 was used as a fungal indicator pathogen, *Staphylococcus aureus* ATCC 25923, *Enterococcus faecalis* ATCC 29212, *Bacillus subtilis* ATCC 6633, *Pseudomonas aeruginosa* ATCC 27853, and *Mycobacterium smegmatis* ATCC 700084 were used as the bacterial indicator pathogen. As a result, compounds **1**–**4** exhibited moderate anti-microbial activity to the *Bacillus subtilis, Staphylococcus aureus*, and *Enterococcus faecalis* but did not show any activity to *Candida albicans*, *Pseudomonas aeruginosa*, and *Mycobacterium smegmatis* (Table 5).

Shellmycins exhibited cytotoxicity at the micro molarity level against five tumorous human cell lines (Table 6). The resazurin-based cell viability assays showed that the four compounds all inhibited cell growth for both 24 h and 72 h treatment. In general, compound **1**, **2**, and **4** showed higher cytotoxicity than compound **3**, with the IC_50_ ranging from 0.69 µM to 3.11 µM at 72 h. In addition, certain types of tumor cells like A375, HepG2, and HT29 are more sensitive to shellmycins. Since compound **3** and **4** are enantiomers, the different anticancer activity of **3** and **4** lead to a conclusion that the stereochemistry of the C-14 is an important factor for the bioactivity of shellmycins. The strong anticancer activity of **1**, **2**, and **4** indicates the potential of such compounds as a template or scaffold to develop structurally related anticancer drugs.

### 2.4. Proposed Biosynthesis Pathway

**Figure 8:**
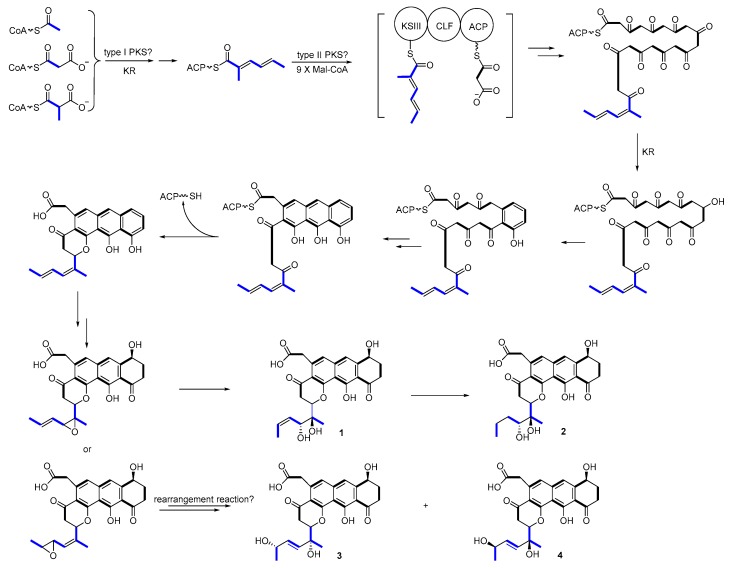
Proposed biosynthetic pathway.

Upon comparing the antibiotic and anticancer activities of different shellmycins, the SAR results then will be used to guide biosynthetic engineering efforts. The remarkable activity of compound **1**, **2**, and **4** implies the importance of the stereochemistry of the side chains. The biosynthesis, incorporation, and post-modification of the side chain, therefore, are significant in the following study. In our proposed biosynthetic pathway of shellmycins, the aromatic polycyclic skeleton of shellmycin suggests that a type-II PKS should be involved in the biosynthesis [29,30]. The methylated 6-carbon side chain of shellmycin is proposed to be synthesized via a bacterial type I PKS, incorporating acetyl-CoA, malonyl-CoA, and methyl-malonyl-CoA sequentially. Here we predict that the nascent product of this type I PKS is methyldiene-S-ACP, which may help to allow a series of epoxidations and rearrangements to take place (Figure 8). This hexenoate starter unit will then be loaded to the type II PKS to form the final tetrahydroanthra-γ-pyrone core after nine rounds of chain elongation (Figure 8). According to the research done by Thorson and co-workers on the biosynthesis of hedamycin, which shares the similar core structure with shellmycin, they suggested that the unusual hexenoate starter unit is recognized by a ketosynthase (KSIII), and loaded onto the PKS by a putative acyl carrier protein (ACP) [31]. In our case, a gatekeeper KSIII and ACP might also be good indications for unusual starter unit incorporation (Figure 8). Oxidative tailoring reactions will then take place on both tetrahydroanthra-γ-pyrone core and side chains. Epoxidation followed by hydrolysis of the diene side chain would form compound **1**, with further reduction in the case of compound **2**. An enzymatic catalyzed rearrangement hydrolysis of the mono-epoxide will result in compounds **3** and **4**. In summary, there are many very intriguing aspects in the proposed biosynthetic mechanism. Mechanistic details will be revealed in the further studies.

## 3. Materials and Methods

### 3.1. General Experimental Procedures

NMR spectra were recorded on a Bruker Avance DRX 500 MHz NMR spectrometer (Bruker Daltonics Inc., Billerica, Massachusetts) with tetramethylsilane (TMS) as an internal standard. HR-ESIMS were measured on an LTQ-Orbitrap XL. UV spectra were recorded on a Persee TU-1810 spectrometer (Beijing Persee General Instrument Co. Ltd., Beijing, China). Optical rotations were measured on SGW-2 digital polarimeter (Shanghai Precision Instrument Co., Ltd., Shanghai, China) at 25 °C. HPLC separations were mainly performed on two HPLC systems: Agilent 1260 instrument equipped with Phenomenex Kinetex PS C18 column (3.5 µm, 10 × 150 mm). Waters PrepLC 150 HPLC System equipped with Waters 996 Photodiode Array Detector using Phenomenex Kinetex PS C18 column (5 µm, 20 × 150 mm). Sephadex LH-20 was obtained from the GE Amersham Biosciences (Piscataway, New Jersey, USA). Reversed-phase C-18 silica gel for column chromatography was purchased from Merck (Darmstadt, Germany). Silica gel GF_254_ for thin-layer chromatography (TLC) was obtained from Qingdao Marine Chemical Ltd. (Qingdao, China).

### 3.2. Biological Materials

The *Streptomyces* sp. shell-016 was isolated from the shell sediment of the Binzhou Shell Dike Island and Wetland National Nature Reserve, China (38°13′17″ N, 117°57′29″ E). The identification of the actinomycete strain was based on 16S rRNA gene sequence analysis. The DNA preparations were used as a template for partial 16S rRNA gene PCR amplification applying the universal primers (27f: 5′-AGAGTTTGATCMTGGCTCAG-3′, 1492r: 5′-TACGGTTACCTTGTTACGACTT-3). The reaction mixture (50 µL) includes 10–100 ng genomic DNA, 20 pmol of each universal primer, 25 µL GoTaq Master Mixes (Promega Corporation, Madison, WI, USA). The PCR cycling program was as follows: initial denaturing for 2 min at 95 °C, 30 cycles of denaturation for 30 s at 95 °C, annealing for 30 s at 58 °C, extension for 60 s at 72 °C, and final extension for 5 min at 72 °C. The sequences of 16S rRNA were assigned into taxonomic ranks (genus) using Ribosomal Database Project (RDP) Classifier. The 16S rRNA gene sequence of the strain displayed 98% similarity with type strains of *Streptomyces achromogenes* subsp. rubradiris strain NBRC 14,000 (NR_112428.1) and *Streptomyces vellosus* strain HR 29 (KT438921.1).

### 3.3. Fermentation and Extraction

The spores of *Streptomyces* sp. shell-016 were cultured with ISP2 agar medium (yeast extract 4.0 g, malt extract 10.0 g, glucose 4.0 g, agar 15 g, dd H_2_O 1000 mL, pH 7.2) in Petri dishes as the seed culture. The fermentation (10 L) was performed on ISP3 agar medium (oatmeal 20 g, saline salt 1 mL (saline salt contained FeSO_4_·7H_2_O 0.1 g, ZnSO_4_·7H_2_O 0.1 g, MnCl_2_·4H_2_O 0.1 g, dd H_2_O 100 mL), dd H_2_O 1000 mL, agar 15 g, pH 7.2) for 12 d at 28 °C in Petri dishes. To extract the metabolites, the culture agar was diced and extracted overnight three times with ethyl acetate: methanol: acetic acid (80:15:5, v/v) at room temperature. After removing the organic solvents under vacuum, the crude extract was partitioned between purified water and ethyl acetate (1:1, v/v). Then the ethyl acetate extract was dried with sodium sulfate (anhydrous) and partitioned between methanol and petroleum ether (1:1, v/v). The methanol layer was concentrated under vacuum to obtain the defatted methanol extract (3.9 g).

### 3.4. Purification of the Compounds

The methanol extract (3.9 g) was fractionated by MPLC (200 g RP-18 silica gel; 30%, 50%, 70%, and 100% methanol, 1 L each, respectively). According to the HPLC detection results, Fr. 3–5, Fr. 9–12, and Fr. 13–15 were combined and marked as Fr. A and Fr. B. Fr. A was subject to Sephadex LH-20 (60 g; methanol) to obtain Fr. A1. Fr. A1 was purified by reversed phase HPLC (Agilent 1260 instrument equipped with Phenomenex Kinetex PS C18 column, 3.5 µm, 10 × 150 mm) eluted with 20% acetonitrile at a flow rate 3.2 mL/min to obtain **3** (*t_R_* = 15.9 min, 2.2 mg) and **4** (*t_R_* = 18.6 min, 1.5 mg).

Fr. B was subject to Sephadex LH-20 (60 g; methanol) to obtain Fr. B1 and Fr. B2. Fr. B1 was purified by reversed phase HPLC (Waters P150 instrument equipped with Phenomenex Kinetex PS C18 column, 5 µm, 20 × 150 mm) eluted with 25% acetonitrile at a flow rate of 15.0 mL/min to obtain **1** (*t_R_* = 5.1 min, 26.6 mg).

Fr. B2 was purified by reversed phase HPLC (Agilent 1260 instrument equipped with Phenomenex Kinetex PS C18 column, 3.5 µm, 10 × 150 mm) eluted with 35% acetonitrile at flow rate of 3.2 mL/min to obtain **2** (*t_R_* = 5.2 min, 2.3 mg).

### 3.5. Spectral Data of the Compounds

**Shellmycin A** (**1**): yellow solid; [α]${}_{D}^{25}$ −261° (c 0.1, MeOH); UV (MeOH) λmax (log ε): 219 (2.78), 268 (4.36), 390 (1.83) nm; NMR data: Table 1; HR-MS *m*/*z* 469.1493, C_25_H_24_O_9_ (calcd for C_25_H_25_O_9_^+^, [M + H]^+^, 469.1493).

**Shellmycin B** (**2**): yellow oil; [α]${}_{D}^{25}$ −235° (c 0.1, MeOH); UV (MeOH) λmax (log ε): 218 (2.85), 267 (4.21), 385 (2.13) nm; NMR data: Table 2; HR-MS *m*/*z* 471.1648, C_25_H_26_O_9_ (calcd for C_25_H_27_O_9_^+^, [M + H]^+^, 471.1650).

**Shellmycin C** (**3**): yellow solid; [α]${}_{D}^{25}$ −98° (c 0.1, MeOH); UV (MeOH) λmax (log ε): 219 (2.35), 266 (4.20), 388 (1.68) nm; NMR data: Table 3; HR-MS *m*/*z* 469.1491 C_25_H_24_O_9_ (calcd for C_25_H_25_O_9_^+^, [M + H]^+^, 469.1493).

**Shellmycin D** (**4**): yellow solid; [α]${}_{D}^{25}$ −223° (c 0.1, MeOH); UV (MeOH λmax (log ε): 219 (2.36), 266 (4.16), 388 (1.83) nm; NMR data: Table 4; HR-MS *m*/*z* 469.1494, C_25_H_24_O_9_ (calcd for C_25_H_25_O_9_^+^, [M + H]^+^, 469.1493). 

### 3.6. Computational Chemistry

The systematically conformational searches were performed by the Pcmodel 10.0 software with a simulated annealing method and the MMFF94 force field. The conformers were further optimized by the B97-3C level while using Orca 4.2 software [32]. The 6-31G** basis set is used by default. The theoretical calculations of the ECD data were conducted while using the TDDFT method at the CAM-B3LYP level in MeOH. The ECD spectra were simulated using the Multiwfn 3.6 [33] to get the Boltzmann-averaged ECD spectrum.

### 3.7. Cytotoxicity Assay

The cytotoxicity of the compounds and positive control were evaluated against five tumorous human cell lines including non-small cell lung cancer (H1299, ATCC CRL-5803), malignant melanoma (A375, ATCC CRL-1619), hepatocellular carcinoma (HepG2, ATCC HB-8065), colorectal adenocarcinoma (HT29 ATCC HTB-38), and breast cancer (HCC1937, ATCC CRL-2336). Cancer cells were plated at a density of 1 × 10^4^ cells per well in 96-well plates and incubated at 37 °C, 5% CO_2_ for 24 h in DMEM-FBS medium. Cells were treated with different concentrations of shellmycins. The medium was removed, and 100 µL of resazurin-containing medium was added to each well after 24 h and 72 h. Cells were then incubated for 2 h, and fluorescence was measured (Cytation 3, BioTek) at excitation/emission wavelengths of 560/590 nm. 

### 3.8. Antimicrobial Assay

*Candida albicans* ATCC 18804 used as indicator fungi, while *Staphylococcus aureus* ATCC 25923, *Enterococcus faecalis* ATCC 29212, *Bacillus subtilis* ATCC 6633, *Pseudomonas aeruginosa* ATCC 27853, and *Mycobacterium smegmatis* ATCC 700084 were used as indicator bacteria.

The MIC was determined by using a broth microdilution assay modified from the method. *Candida albicans* ATCC 18804 was grown in YPD broth and bacteria were grown overnight in LB broth and diluted to approximately 4 × 10^5^ CFU/mL then added 90 µL to the sterile 96-well polypropylene microtiter plates. Two-fold serial dilutions of compounds **1**–**4** in DMSO were added to the 96-well plates with concentrations ranging from 320 to 2.5 µg/mL. Plates were incubated at 37 °C overnight then read at 600 nm in a microplate reader.

## 4. Conclusions

In conclusion, four novel tetrahydroanthra-γ-pyrone compounds named shellmycin A–D were isolated from the marine *Streptomyces* sp. shell-016. The structures of these four compounds were established by interpretation of 1D and 2D NMR, high-resolution ESI-MS data, and single crystal X-ray diffraction. An interesting fact was **3** and **4** are a pair of stereoisomers and their biological activity was significantly different. This study provides not only a valuable subject for future research on the structure–activity relationship and biosynthesis, but also an impetus for anti-cancer drug development.

## Figures and Tables

**Table 1:** NMR spectroscopy data (MeOD-*d*_4_) for compound **1**.

Pos.	δ_H_ (mult., *J* = Hz)	δc	^1^H-^1^H COSY	HMBC
2		174.9s		
3	6.73 (s)	111.6d		C-14, C-2, C-4, C-4a
4		182.1s		
4a		120.6s		
5		138.0s		
6	7.47 (s)	129.9d		C-13, C-12a, C-7, C-4a, C-6a
6a		142.6s		
7	7.43 (s)	117.4d		C-8, C-6, C-11a, C-6a, C-11
7a		147.8s		
8	4.95 (dd, 7.5, 3.8)	69.0d	H-9	C-10, C-7, C-7a
9	2.18, 2.37 (m)	32.7t	H-8, H-10	C-10, C-8, C-7a, C-11
10	2.81, 3.00 (dq, 17.6, 4.6)	36.8t	H-9	C-9, C-8, C-11
11		207.0s		
11a		113.3s		
12		165.8s		
12a		114.7s		
12b		159.1s		
13	4.16,4.26 (m)	42.9t		C-13-COOH, C-5, C-4a, C-6
13-COOH		175.7s		
14		78.1s		
15	1.55 (s)	24.0q		C-3, C-2, C-16
16	5.28 (d, 10.5)	71.5d	H-17	C-3, C-2, C-15
17	5.73 (ddd, 11.7, 10.5, 1.5)	130.0d	H-16, H-18	C-19, C-16, C-14
18	5.88 (dq, 11.7, 6.8)	130.1d	H-17, H-19	C-19, C-16
19	1.92 (dd, 6.9, 1.8)	14.4q	H-18	C-17, C-16

**Table 2:** NMR spectroscopy data (MeOD-*d*_4_) for compound **2**.

Pos.	δ_H_ (mult., *J* = Hz)	δc	^1^H-^1^H COSY	HMBC
2		174.9s		
3	6.72 (s)	111.6d		C-14, C-4a, C-2
4		182.5s		
4a		121.1s		
5		138.4s		
6	7.59 (s)	130.3d		C-13, C-4a, C-7, C-12a
6a		143.0s		
7	7.54 (s)	117.7d		C-8, C-11a, C-6, C-6a
7a		148.3s		
8	5.00 (dd, 8.6, 3.9)	69.3d	H-9	
9	2.21, 2.40 (m)	33.0t	H-8, H-10	
10	2.87, 3.04 (m)	37.0t	H-9	
11		207.3		
11a		113.7s		
12		166.2s		
12a		115.3s		
12b		159.7s		
13	4.26-4.33 (m)	43.3t		C-13-COOH, C-4a, C-6, C-5
13-COOH		176.2s		
14		78.5s		
15	1.65 (s)	24.2q		C-3, C-2, C-16
16	4.19 (dd, 10.2, 1.8)	76.7d	H-17	
17	1.58, 1.69 (m)	34.3t	H-16, H-18	
18	1.47, 1.68 (m)	21.3t	H-17, H-19	
19	1.02 (t, 6.9, 1.8)	15.1q	H-18	C-17, C-16, C-14

**Table 3:** NMR spectroscopy data (MeOD-*d*_4_) for compound **3**.

Pos.	δ_H_ (mult., *J* = Hz)	δc	^1^H-^1^H COSY	HMBC
2		174.8s		
3	6.67 (s)	109.8d		C-14, C-4a, C-2
4		182.6s		
4a		121.0s		
5		138.3s		
6	7.58 (s)	130.4d		C-13, C-4a, C-7, C-12a
6a		143.0s		
7	7.54 (s)	117.6d		C-8, C-11a, C-6, C-6a
7a		148.4s		
8	5.00 (dd, 8.7, 4.0)	69.4d	H-9	C-7, C-7a
9	2.21, 2.40 (m)	33.1t	H-8, H-10	
10	2.87, 3.04 (m)	37.1t	H-9	C-8, C-9
11		207.2s		
11a		113.8s		
12		166.2s		
12a		115.1s		
12b		159.7s		
13	4.28 (m)	43.3t		C-13-COOH, C-4a, C-6, C-5
13-COOH		176.1s		
14		74.8s		
15	1.82 (s)	28.2q		C-17, C-2, C-14
16	6.25 (dd, 15.5, 1.3)	133.9d	H-17	C-14, C-18
17	6.05 (dd, 15.5, 5.8)	136.3d	H-16, H-18	C-14, C-18
18	4.32 (m)	69.5d	H-17, H-19	C-16
19	1.26 (t, 6.9, 1.8)	24.4q	H-18	C-17, C-18

**Table 4:** NMR spectroscopy data (MeOD-*d*_4_) for compound **4**.

Pos.	δ_H_ (mult., *J* = Hz)	δc	^1^H-^1^H COSY	HMBC
2		174.9s		
3	6.67 (s)	109.8d		C-14, C-4a, C-2
4		182.6s		
4a		121.0s		
5		138.3s		
6	7.57 (s)	130.4d		C-13, C-4a, C-7, C-12a
6a		143.0s		
7	7.53 (s)	117.6d		C-8, C-11a, C-6, C-6a
7a		148.4s		
8	5.00 (dd, 8.7, 4.0)	69.4d	H-9	C-7, C-7a
9	2.21, 2.40 (m)	33.1t	H-8, H-10	
10	2.87, 3.04 (m)	37.1t	H-9	C-8, C-9
11		207.2s		
11a		113.8s		
12		166.2s		
12a		115.1s		
12b		159.6s		
13	4.24-4.31 (m)	43.3t		C-13-COOH, C-4a, C-6, C-5
13-COOH		176.1s		
14		74.9s		
15	1.82 (s)	28.3q		C-17, C-2, C-14
16	6.26 (d, 15.5)	133.8d	H-17	C-14, C-18
17	6.05 (dd, 15.5, 5.6)	136.2d	H-16, H-18	C-14, C-18
18	4.33 (m)	69.4d	H-17, H-19	C-16
19	1.25 (t, 6.9, 1.8)	24.4q	H-18	C-17, C-18

**Table 5:** Anti-microbial activity (MIC µg/mL) of compounds **1**–**4**.

Pathogen Strains	MIC (µg/mL)			
	1	2	3	4
*Candida albicans* ATCC 18804	>100	>100	>100	>100
*Staphylococcus aureus* ATCC 25923	3	21	46	16
*Enterococcus faecalis* ATCC 29212	6	13	36	21
*Bacillus subtilis* ATCC 6633	11	26	32	21
*Pseudomonas aeruginosa* ATCC 27853	>100	>100	>100	>100
*Mycobacterium smegmatis* ATCC 700084	>100	>100	>100	>100

**Table 6:** Inhibitory concentration (IC_50_, µM) of compounds **1**–**4** against various tumor cell lines (cis-platinum as positive control).

Compound	IC_50_ (µM)									
	A375		H1299		HepG2		HT29		HCC1937	
	24 h	72 h	24 h	72 h	24 h	72 h	24 h	72 h	24 h	72 h
**1**	5.22	0.69	8.63	1.32	4.22	0.89	4.69	0.85	9.68	2.62
**2**	7.18	0.95	10.3	2.62	5.67	1.35	6.12	1.12	11.6	3.11
**3**	40.3	11.3	>50	26.3	11.3	5.03	13.0	4.33	>50	12.6
**4**	6.04	0.78	10.1	2.15	5.16	1.11	5.37	1.02	10.3	2.89
*cis*-platinum	23.1	9.32	21.2	7.3	>50	18.9	>50	>50	>50	>50

## Supplementary Materials

The following are available online at https://www.mdpi.com/1660-3397/18/1/58/s1, Figure S1: The HR-MS of compound **1**; Figure S2: The ^1^H-NMR of compound **1**; Figure S3: The ^13^C-NMR of compound **1**; Figure S4: The ^1^H-^1^H COSY of compound **1**; Figure S5: The HSQC of compound **1**; Figure S6: The HMBC of compound **1**; Figure S7: The NOESY of compound **1**; Figure S8: The HR-MS of compound **2**; Figure S9: The ^1^H-NMR of compound **2**; Figure S10: The ^13^C-NMR of compound **2**; Figure S11: The ^1^H-^1^H COSY of compound **2**; Figure S12: The HSQC of compound **2**; Figure S13: The HMBC of compound **2**; Figure S14: The NOESY of compound **2**; Figure S15: The HR-MS of compound **3**; Figure S16: The ^1^H-NMR of compound **3**; Figure S17: The ^13^C-NMR of compound **3**; Figure S18: The ^1^H-^1^H COSY of compound **3**; Figure S19: The HSQC of compound **3**; Figure S20: The HMBC of compound **3**; Figure S21: The NOESY of compound **3**; Figure S22: The HR-MS of compound **4**; Figure S23: The ^1^H-NMR of compound **4**; Figure S24: The ^13^C-NMR of compound **4**; Figure S25: The ^1^H-^1^H COSY of compound **4**; Figure S26: The HSQC of compound **4**; Figure S27: The HMBC of compound **4**; Figure S28: The NOESY of compound **4**; 16S ribosomal RNA of *Streptomyces* sp. shell-016, partial sequence; Crystal Structure Reports.
