# Supplementary material for: Shellmycin A–D, Novel Bioactive Tetrahydroanthra-γ-Pyrone Antibiotics from Marine Streptomyces sp. Shell-016
Source: Mar Drugs. 2020 Jan 16;18(1):58. doi: 10.3390/md18010058 (PMC7024178; doi:10.3390/md18010058)
Supplement: Supplementary file 1 [file marinedrugs-18-00058-s001.pdf]

Supplementary Materials

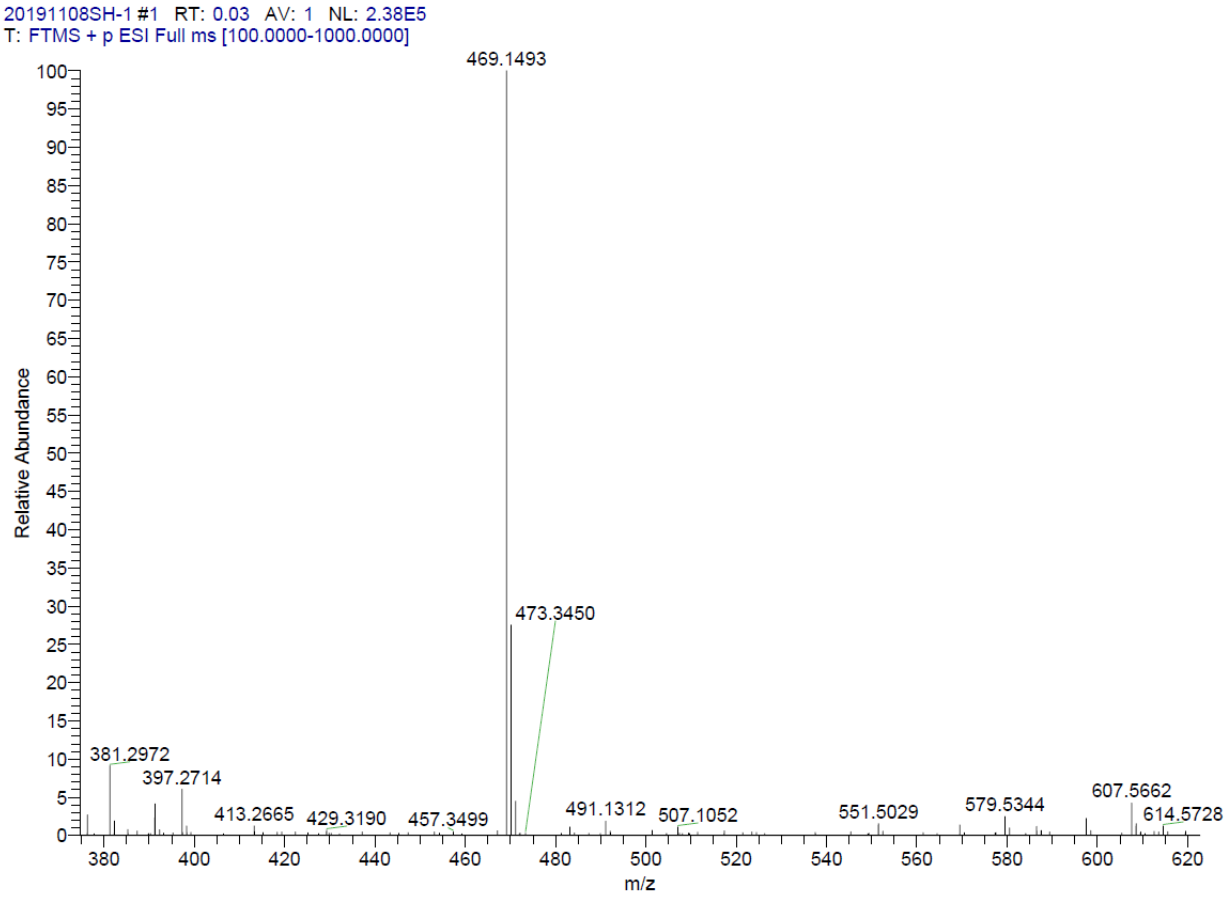

Figure S1. The HR-MS of compound **1**

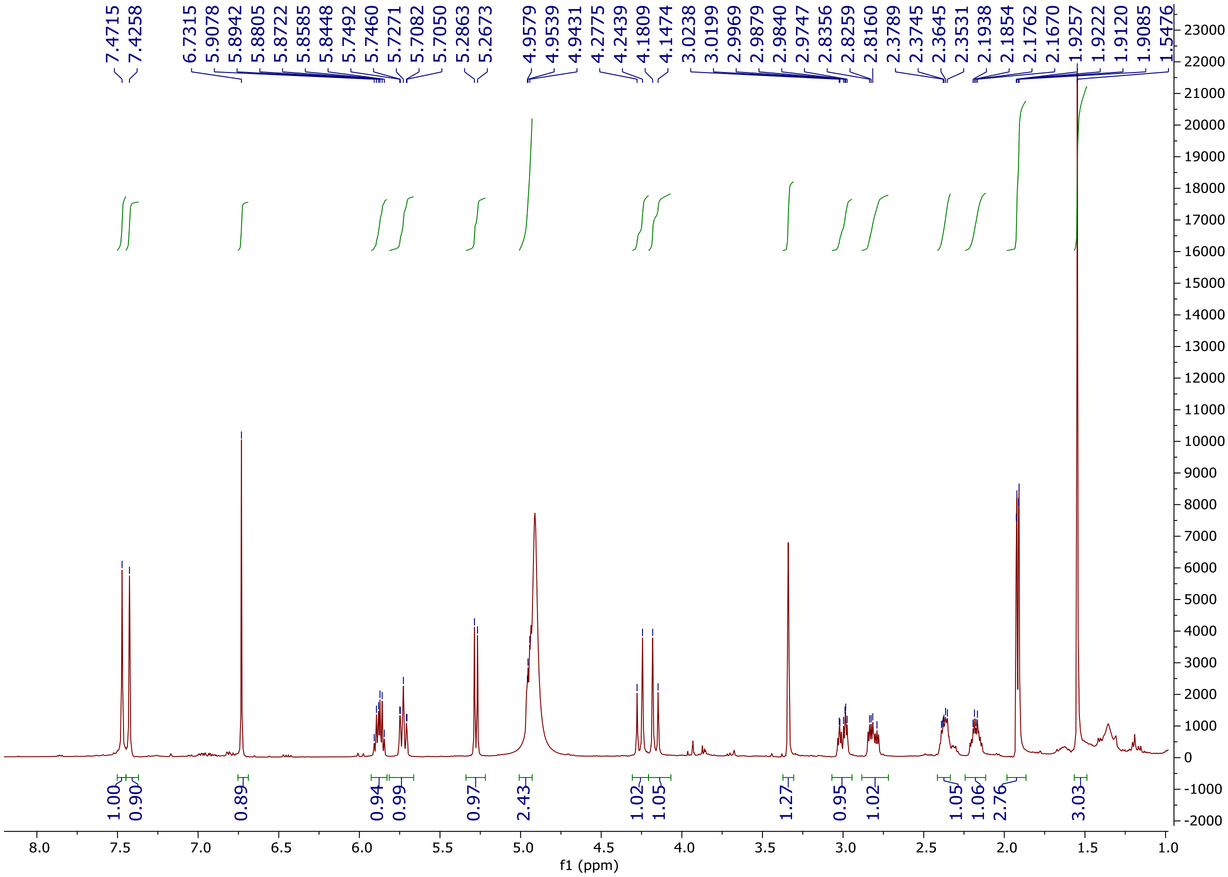

Figure S2. The ^{1}H-NMR of compound **1**

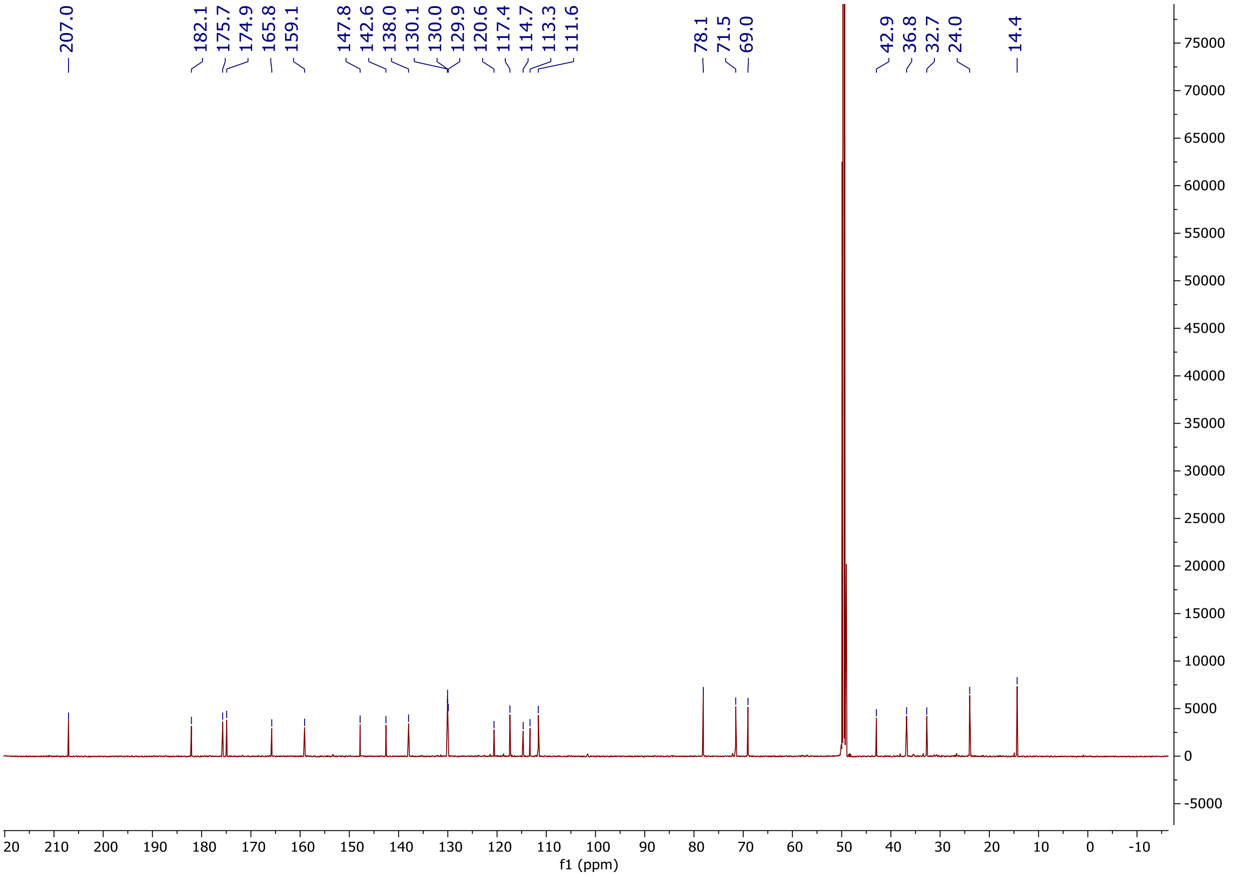

Figure S3. The ^{13}C-NMR of compound **1**

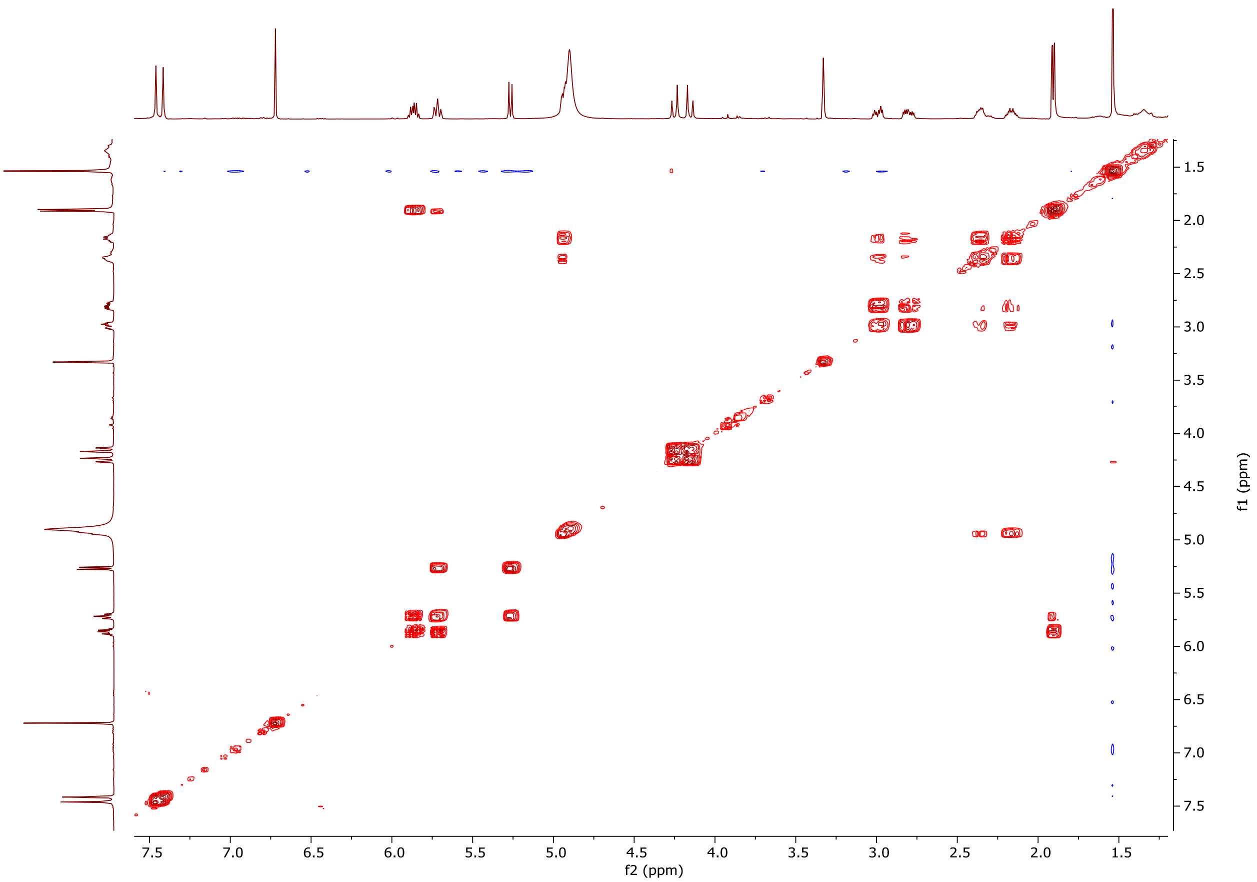

Figure S4. The ^{1}H-^{1}H COSY of compound **1**

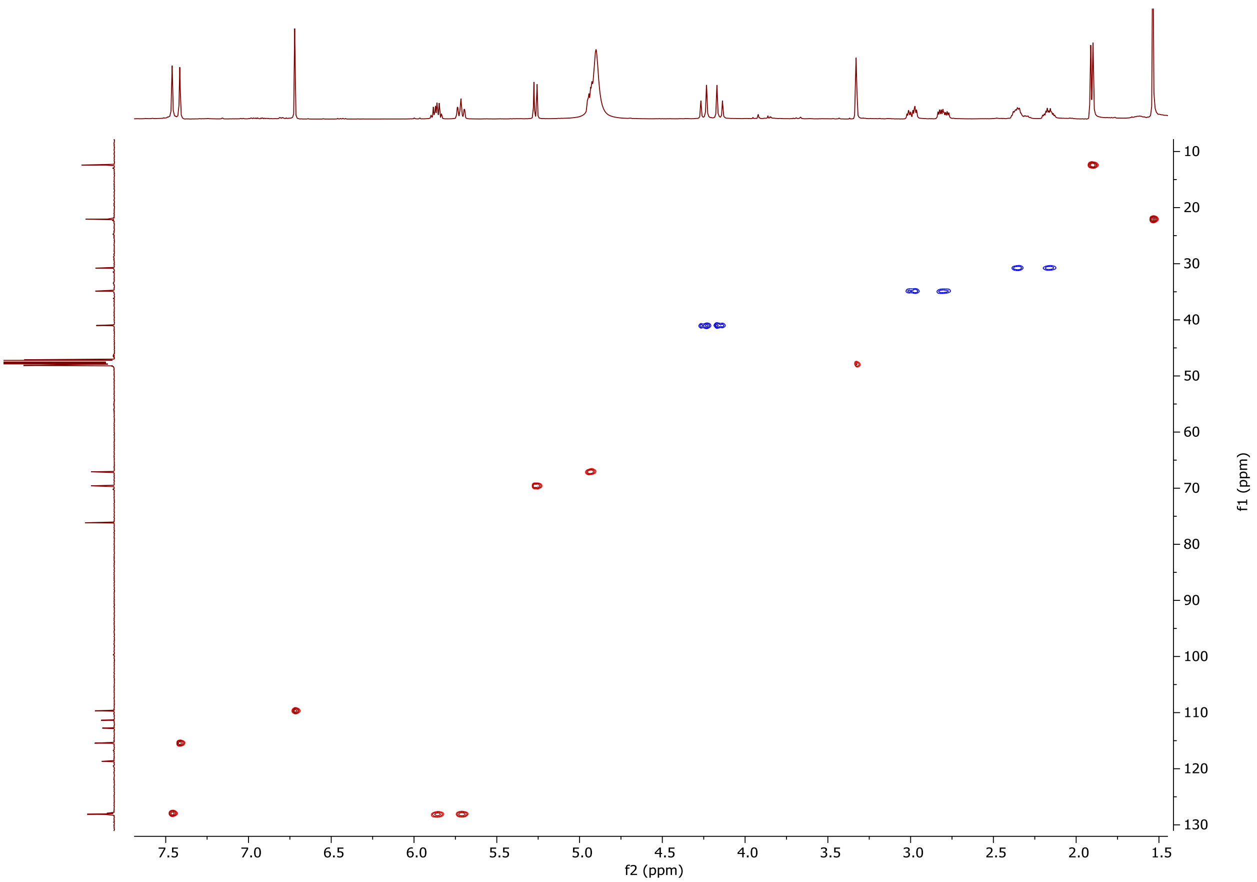

Figure S5. The HSQC of compound **1**

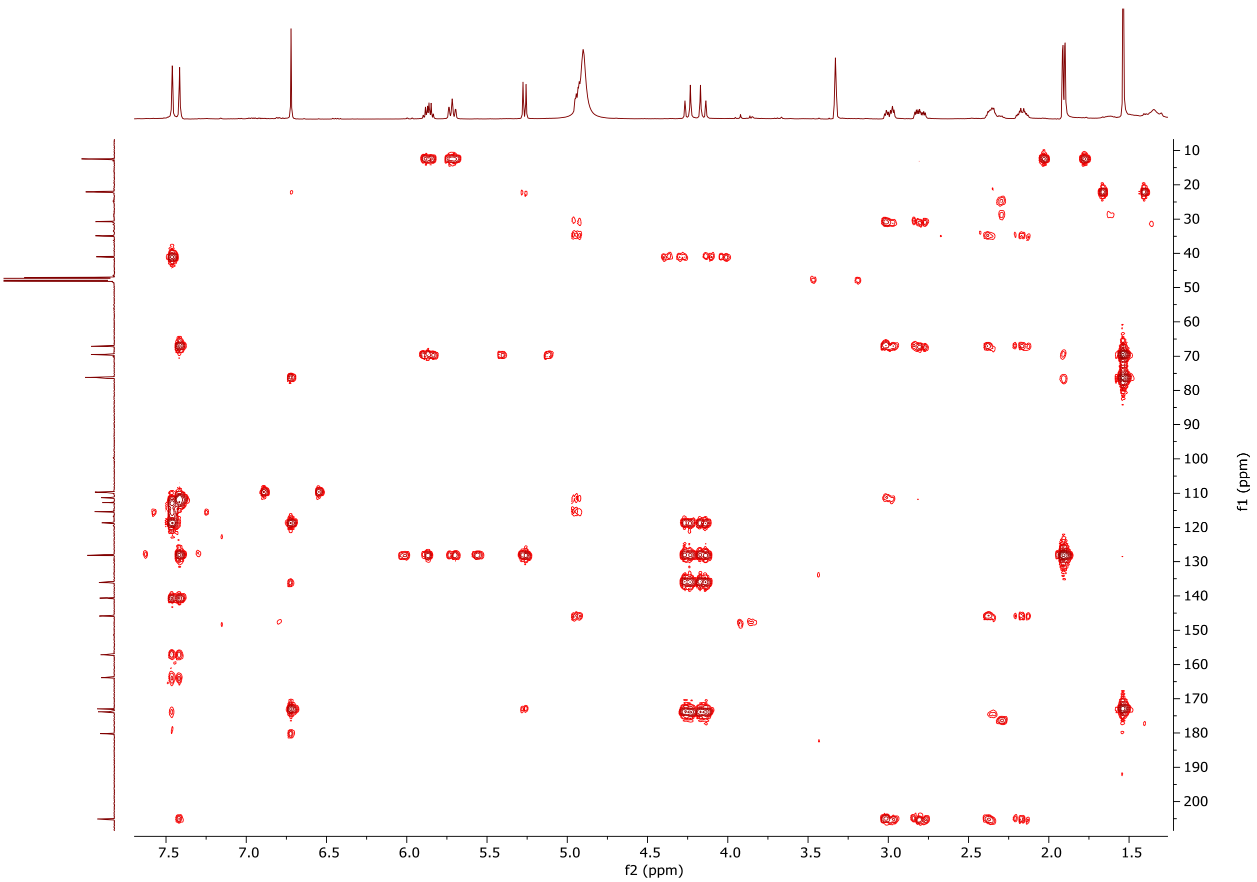

Figure S6. The HMBC of compound **1**

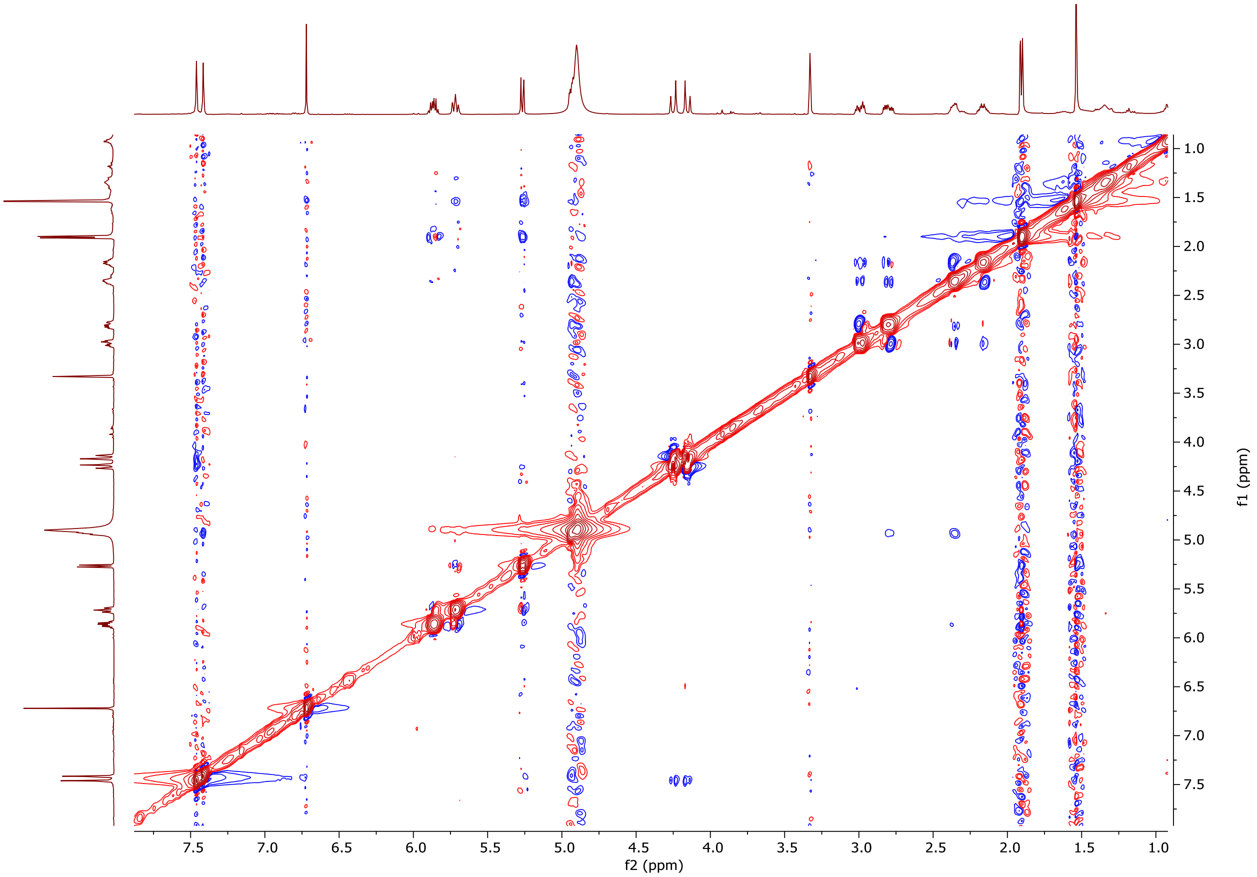

Figure S7. The NOESY of compound **1**

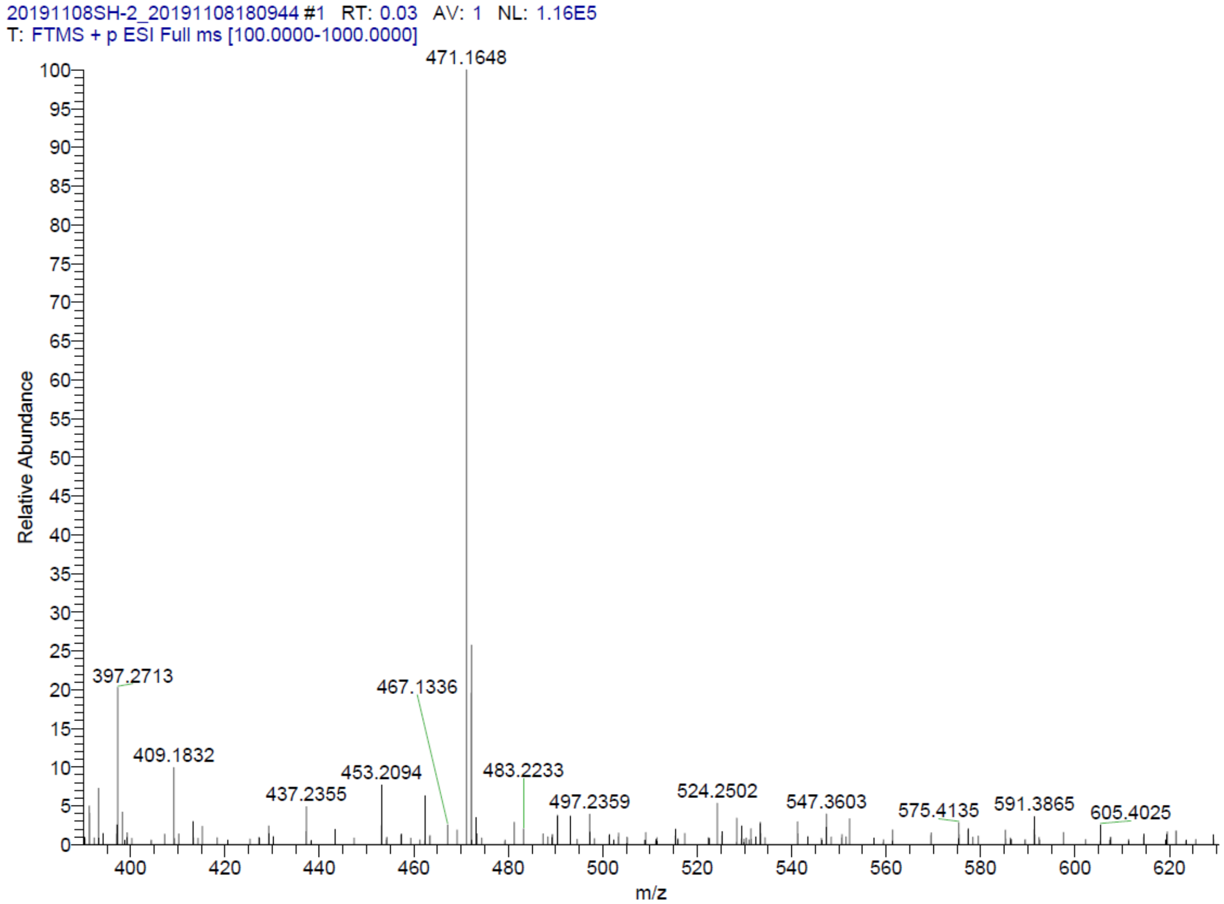

Figure S8. The HR-MS of compound **2**

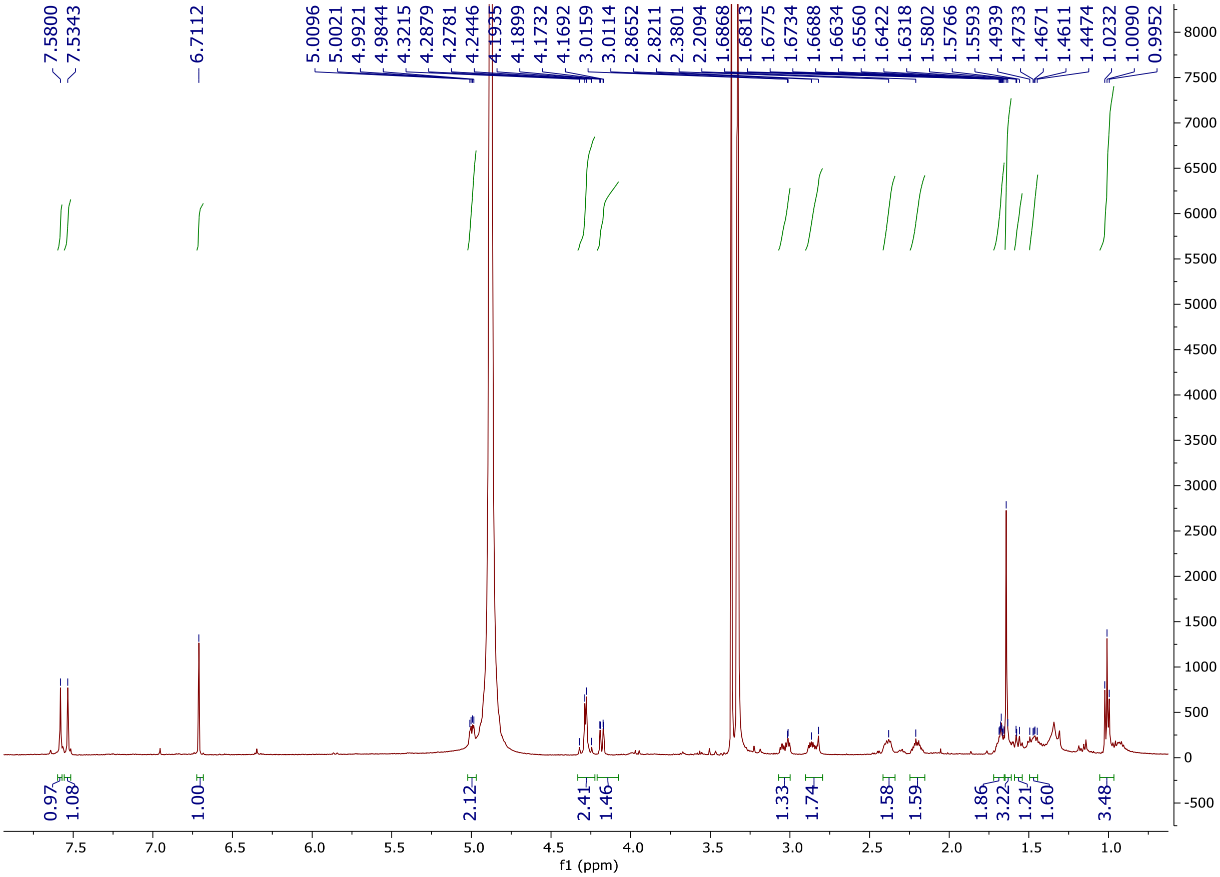

Figure S9. The ^{1}H-NMR of compound **2**

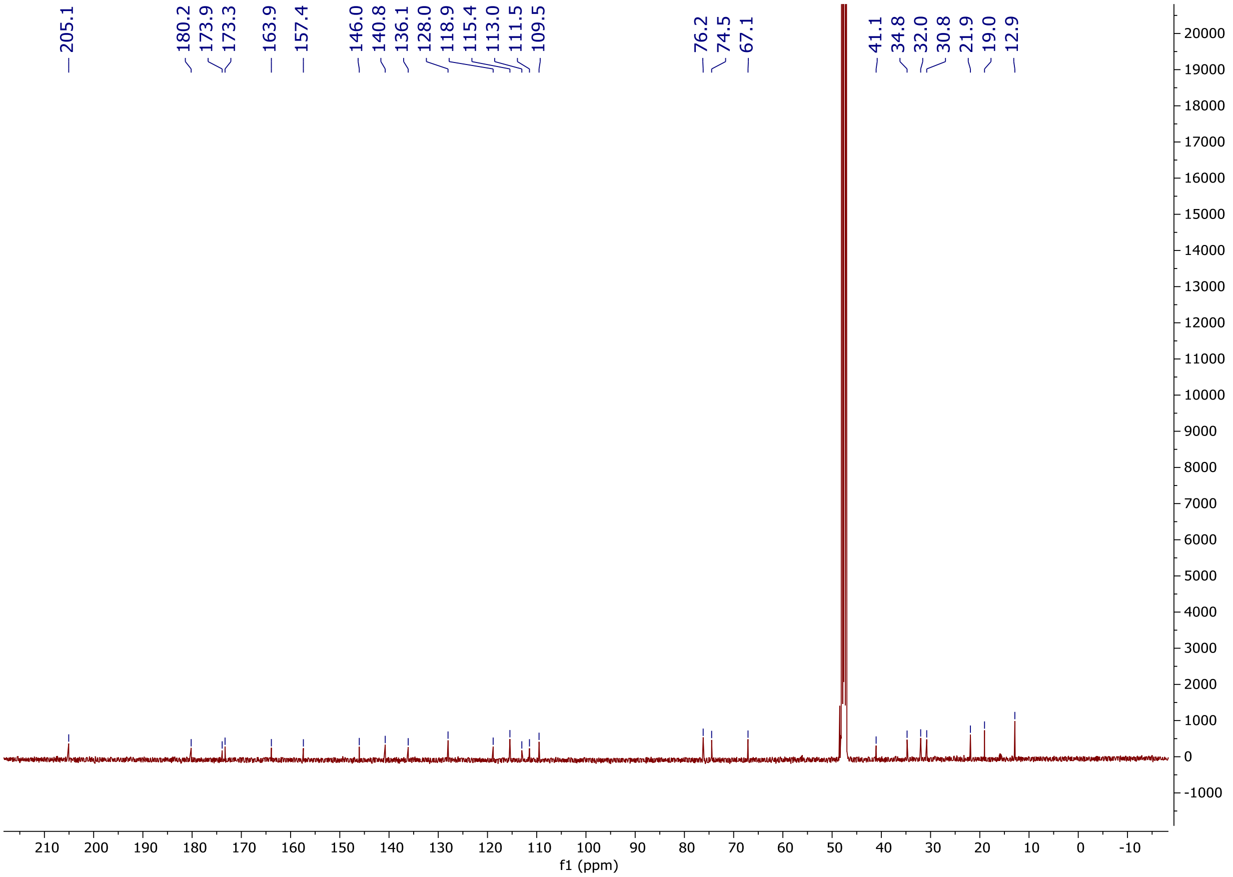

Figure S10. The ^{13}C-NMR of compound **2**

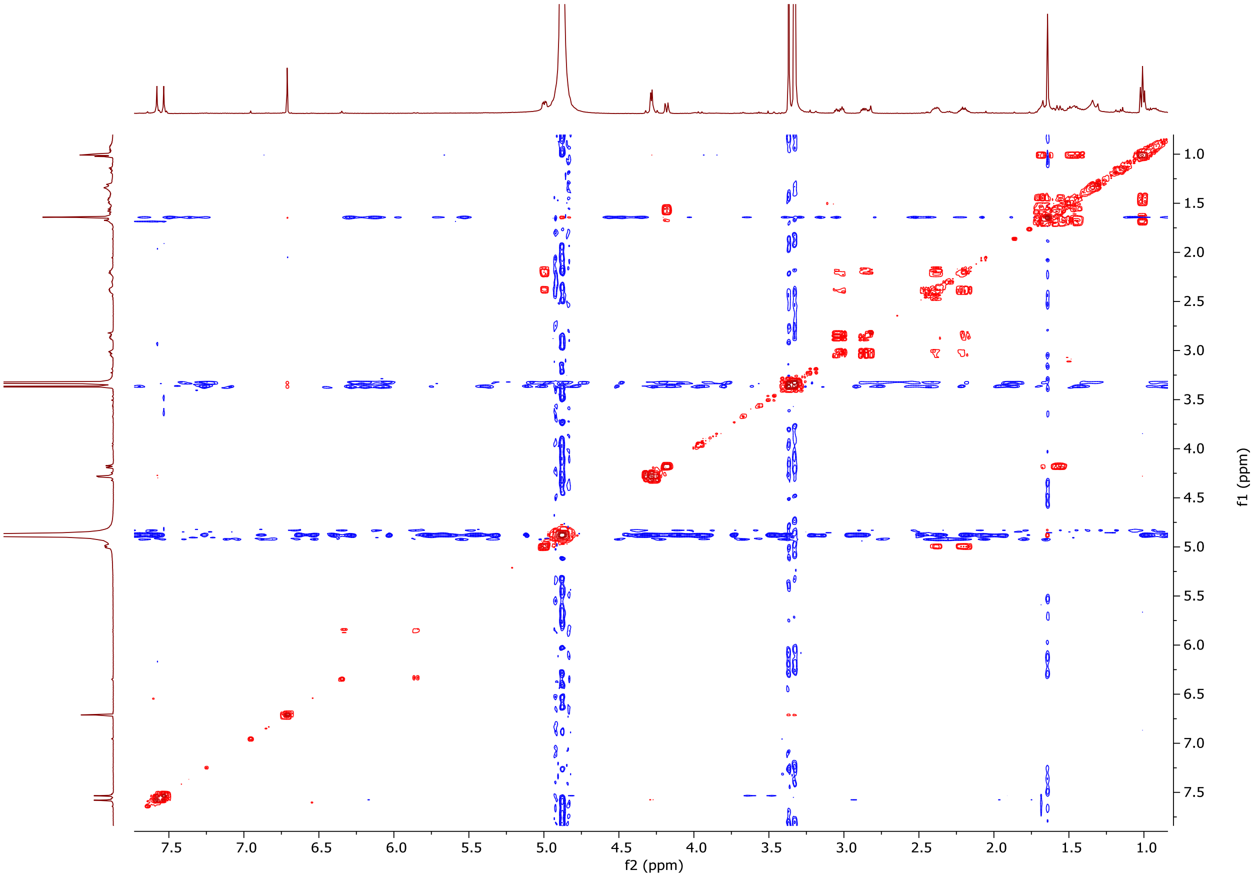

Figure S11. The ^{1}H-^{1}H COSY of compound **2**

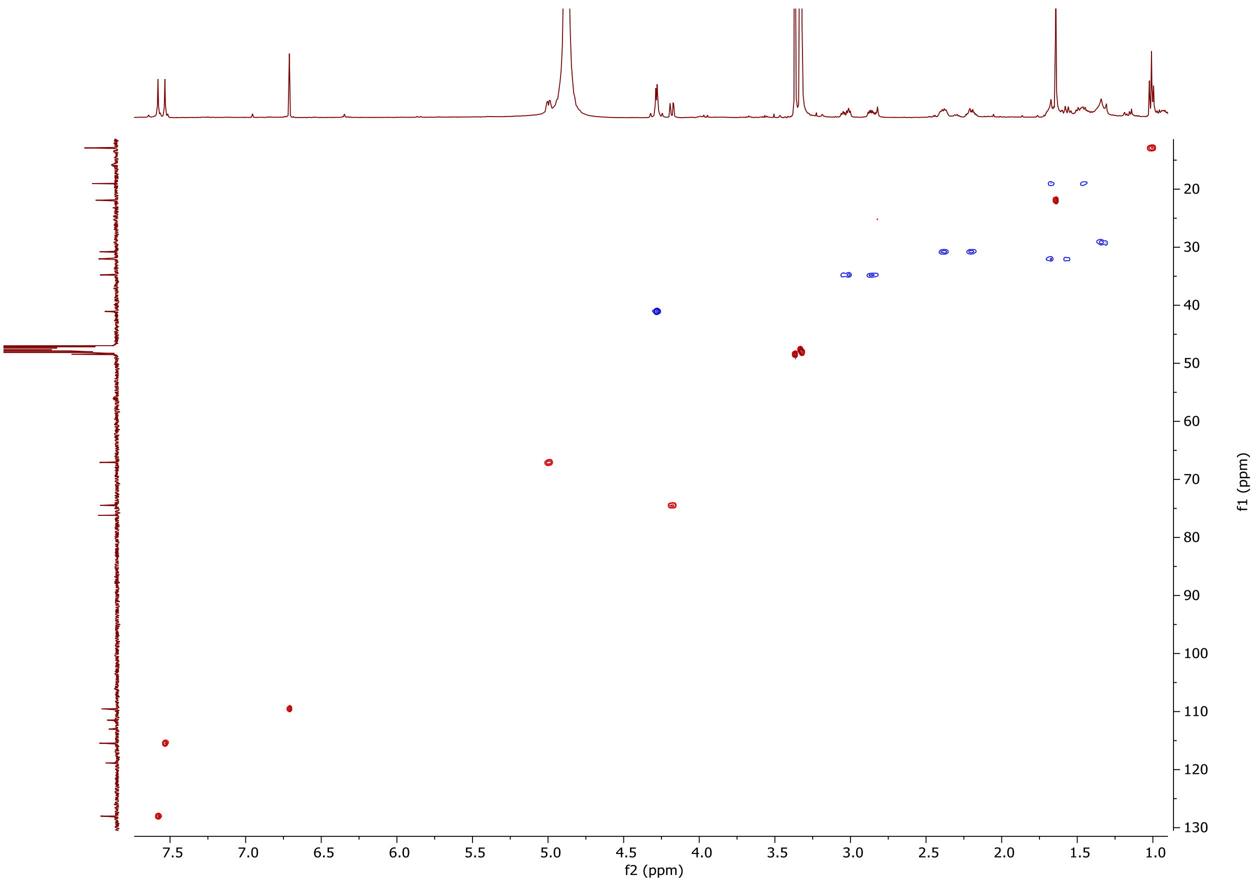

Figure S12. The HSQC of compound **2**

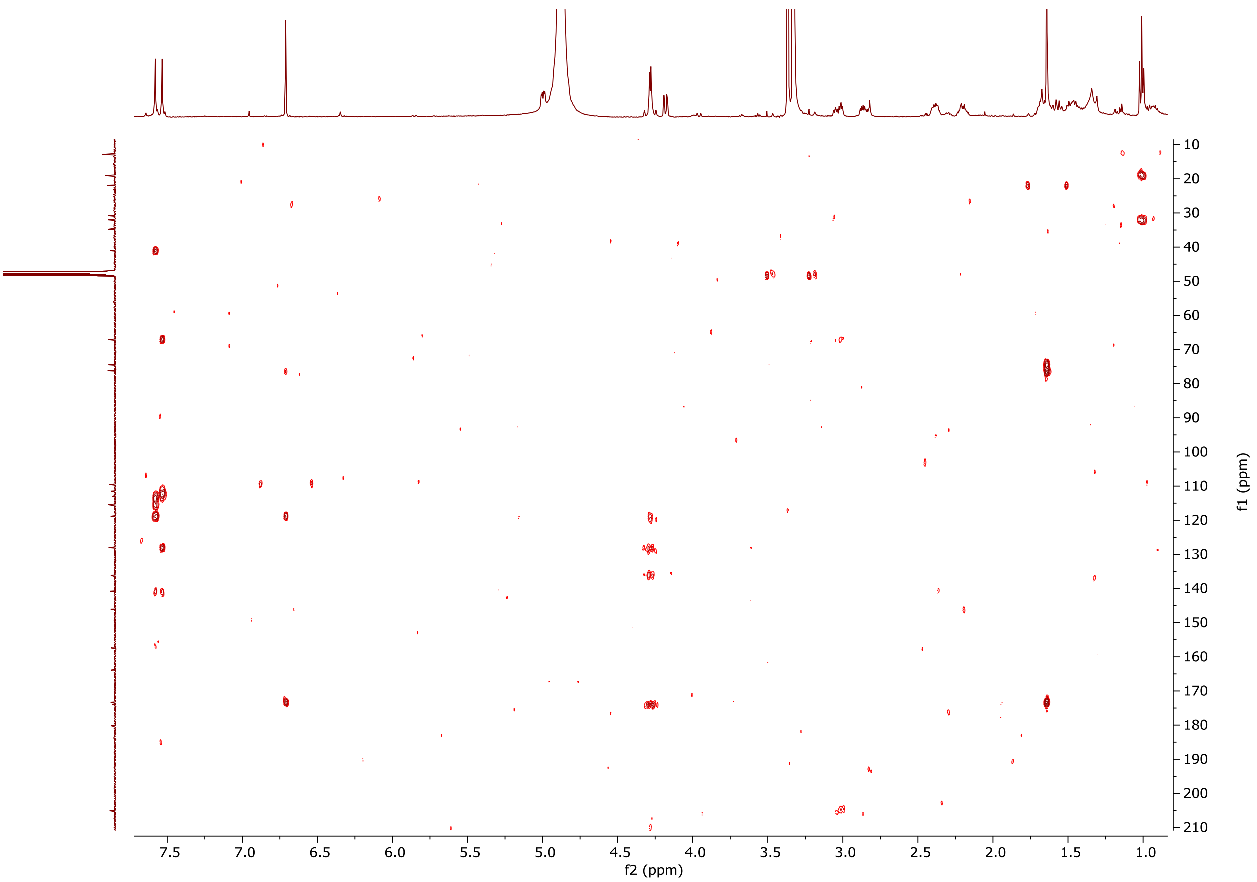

Figure S13. The HMBC of compound **2**

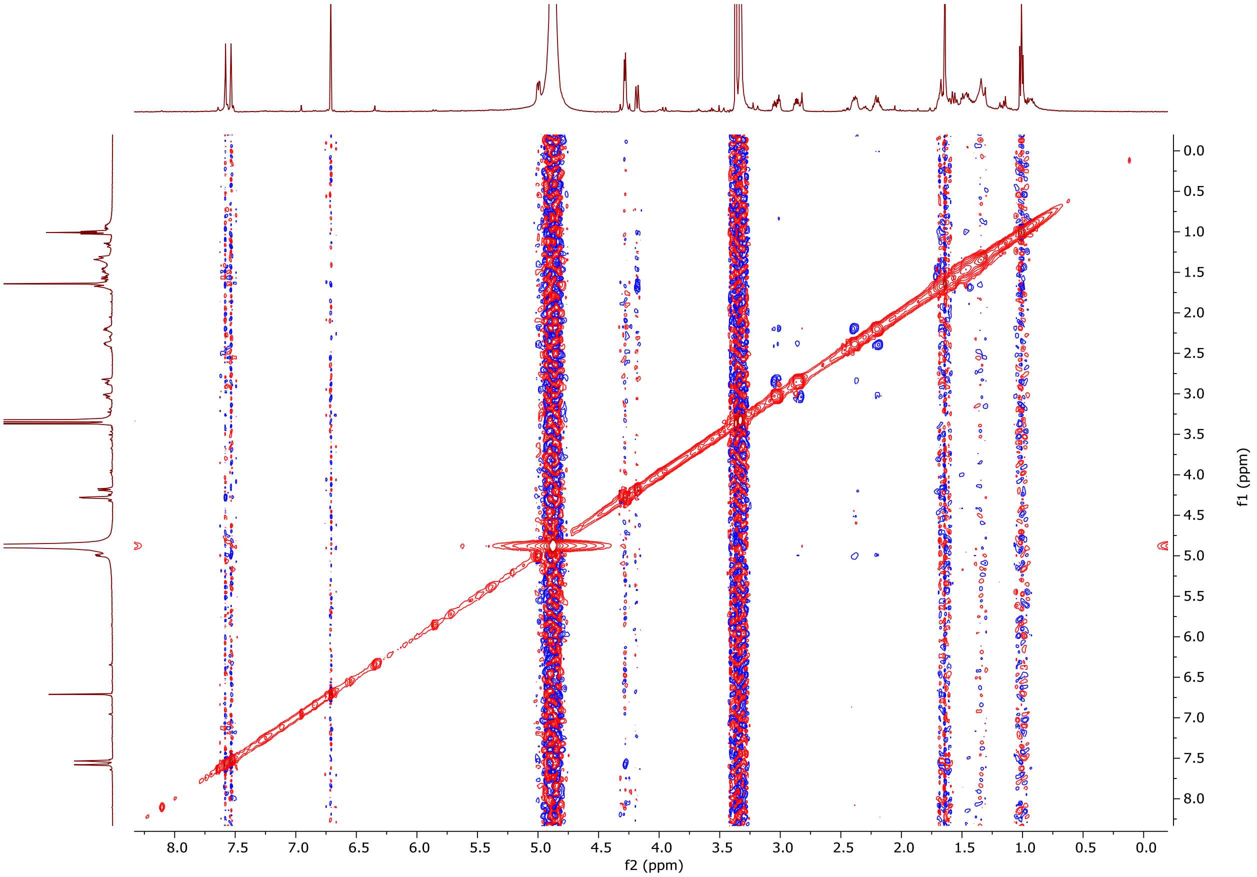

Figure S14. The NOESY of compound **2**

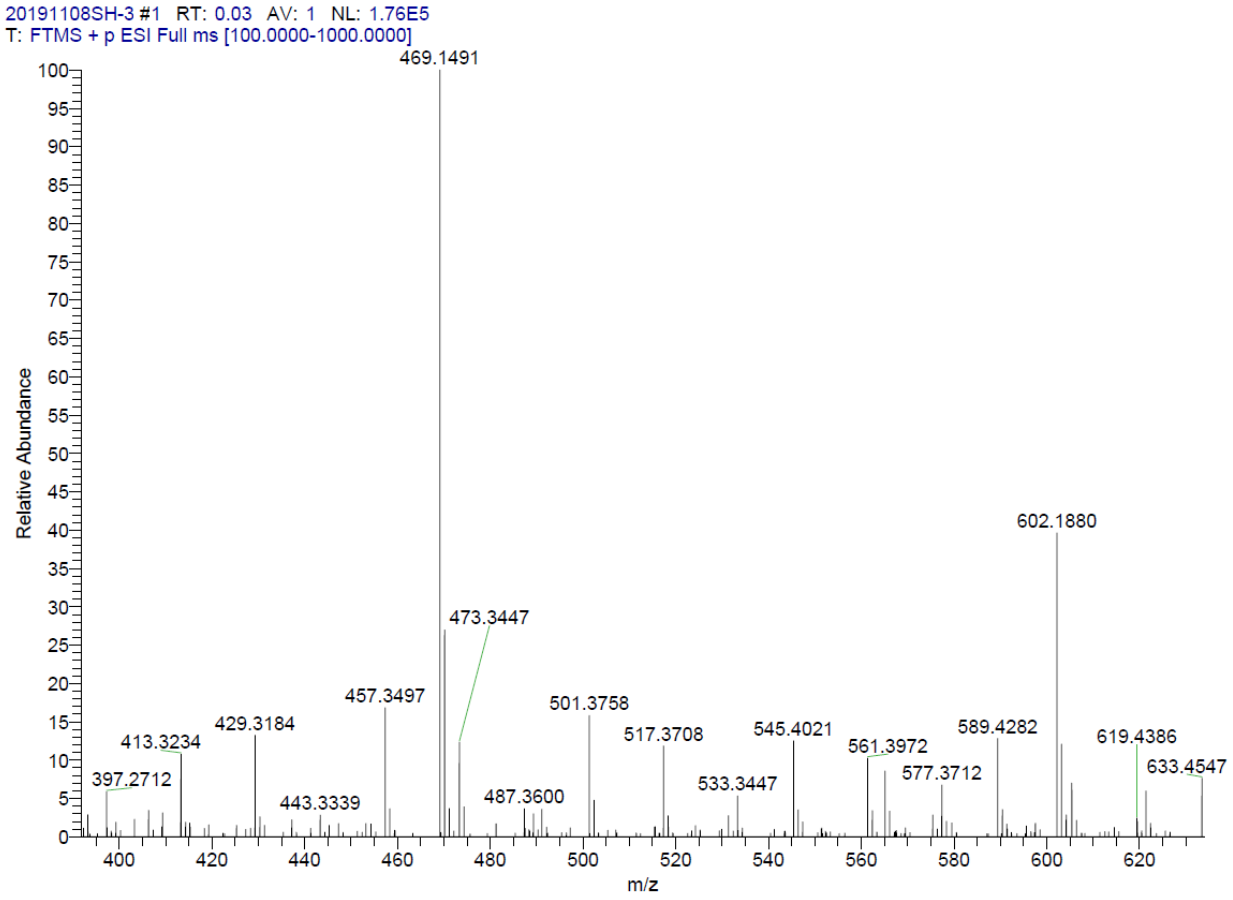

Figure S15. The HR-MS of compound **3**

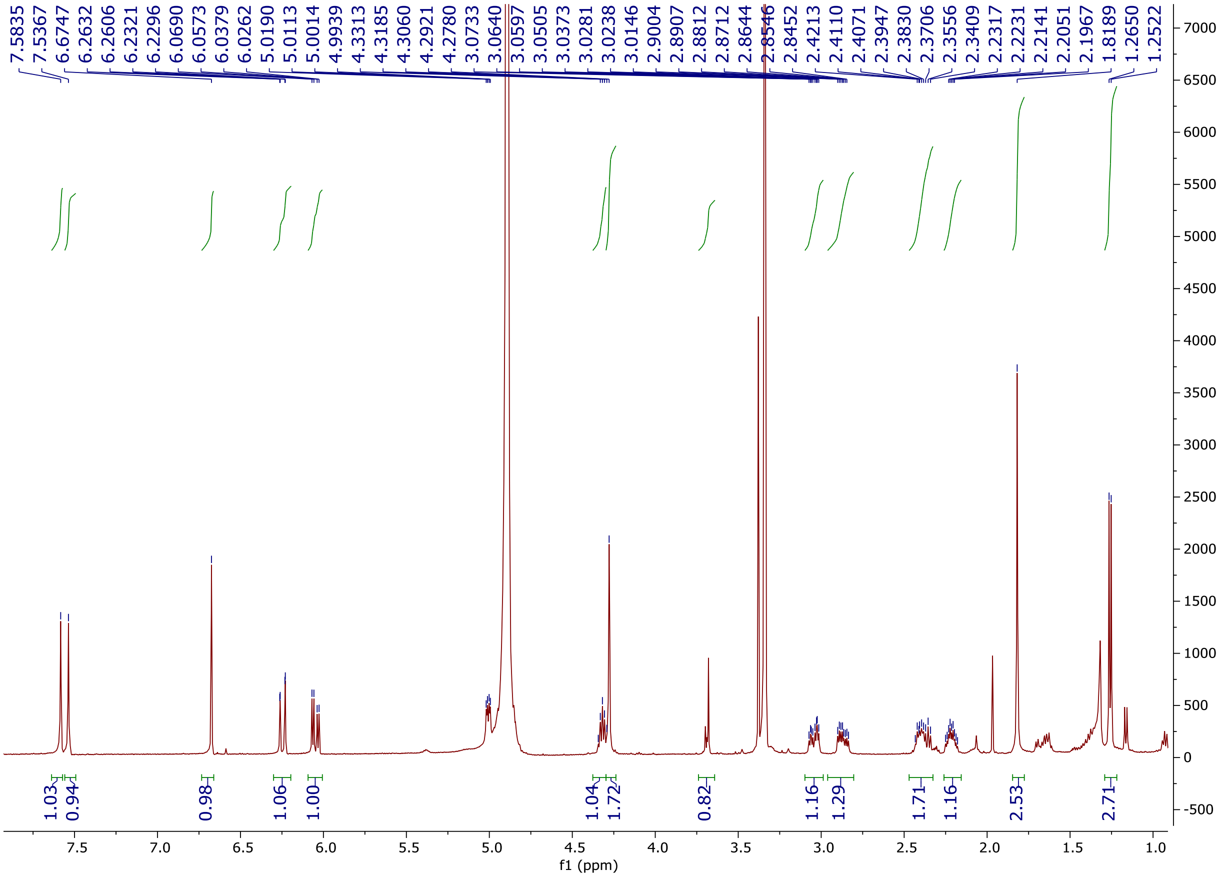

Figure S23. The ^{1}H-NMR of compound **3**

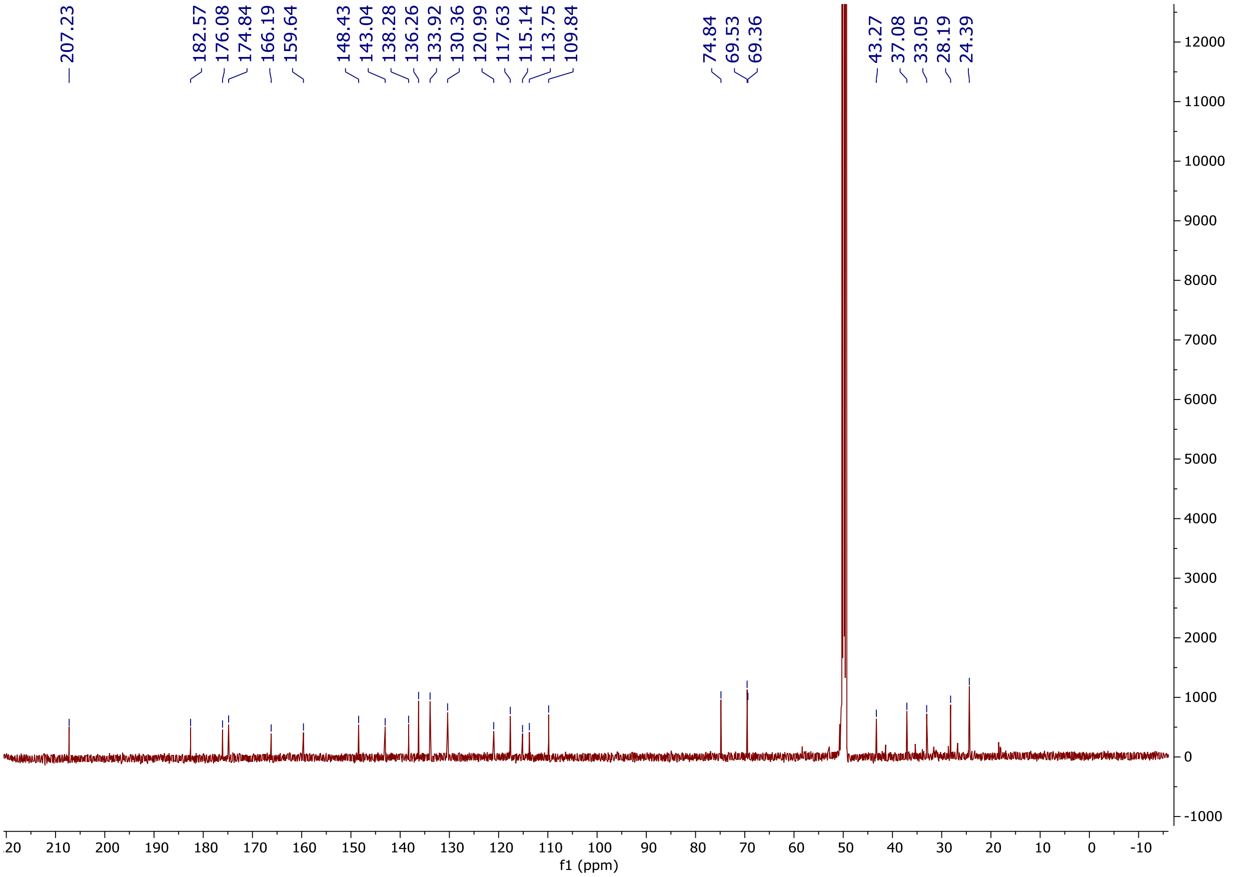

Figure S24. The ^{13}C-NMR of compound **3**

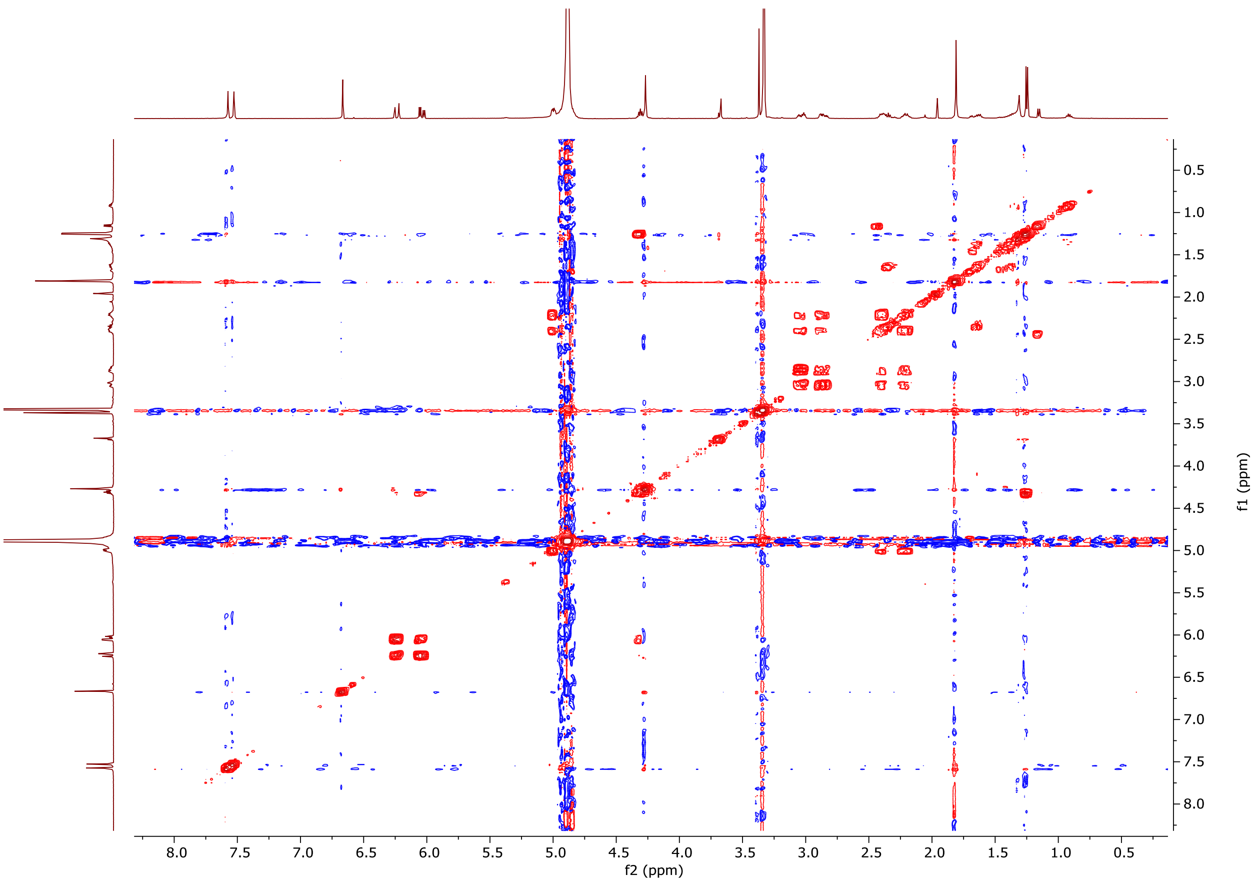

Figure S25. The ^{1}H-^{1}H COSY of compound **3**

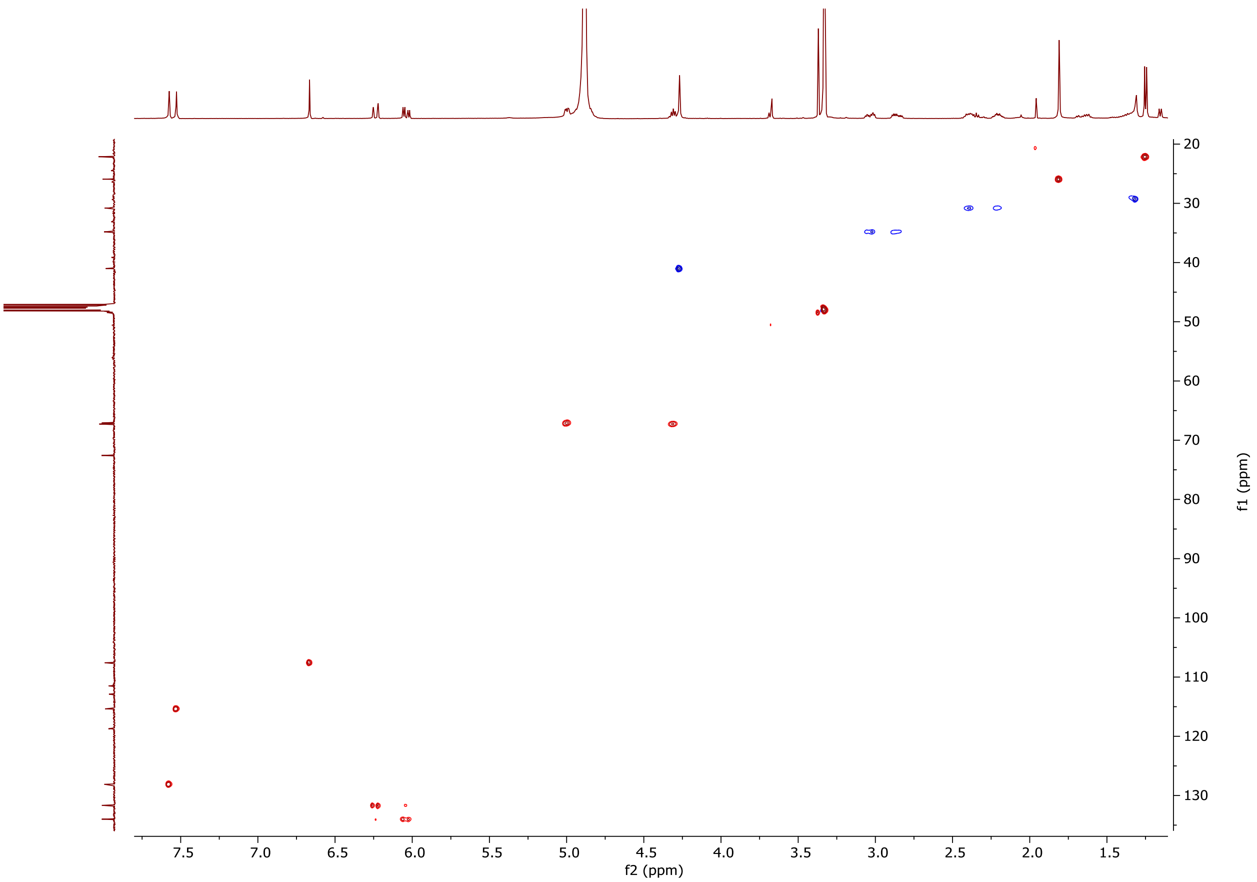

Figure S26. The HSQC of compound **3**

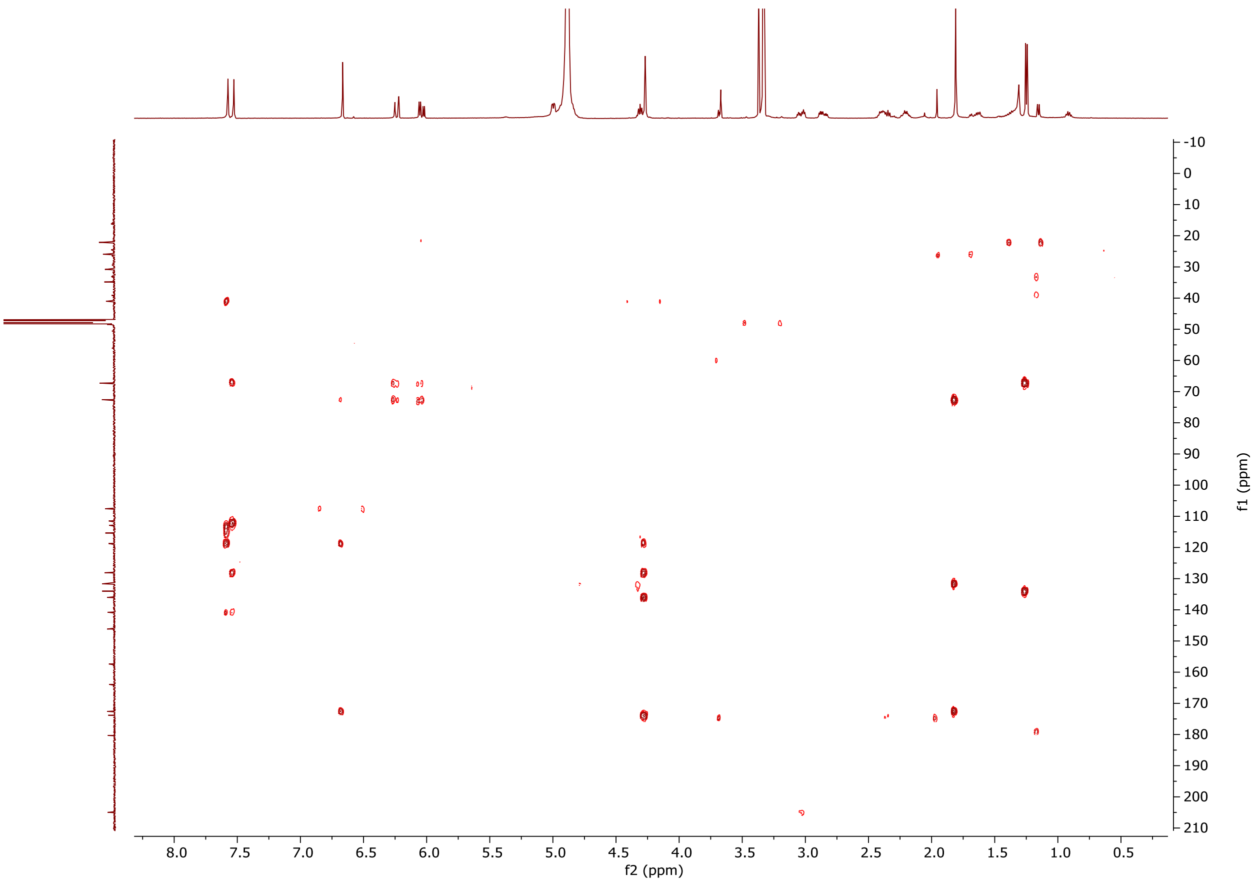

Figure S27. The HMBC of compound **3**

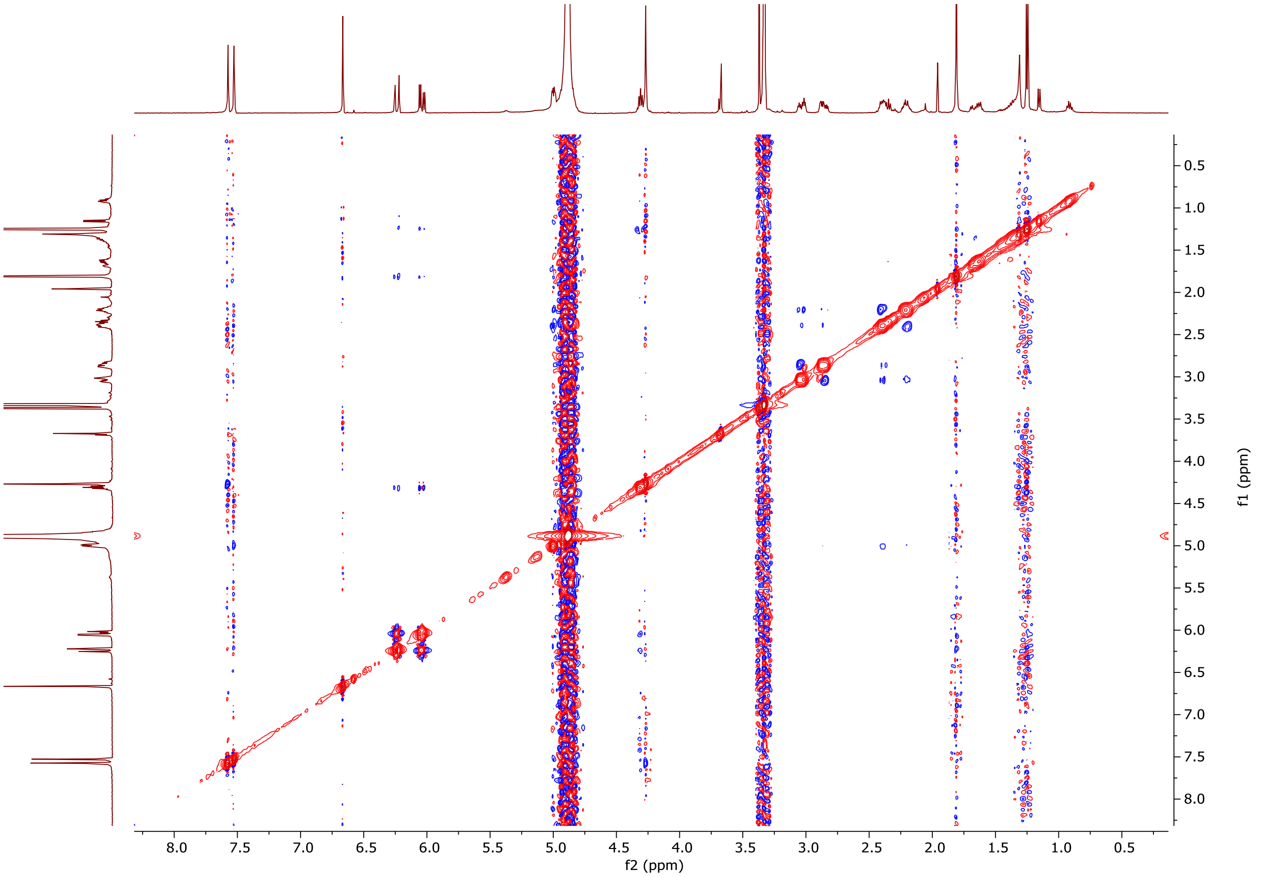

Figure S28. The NOESY of compound **3**

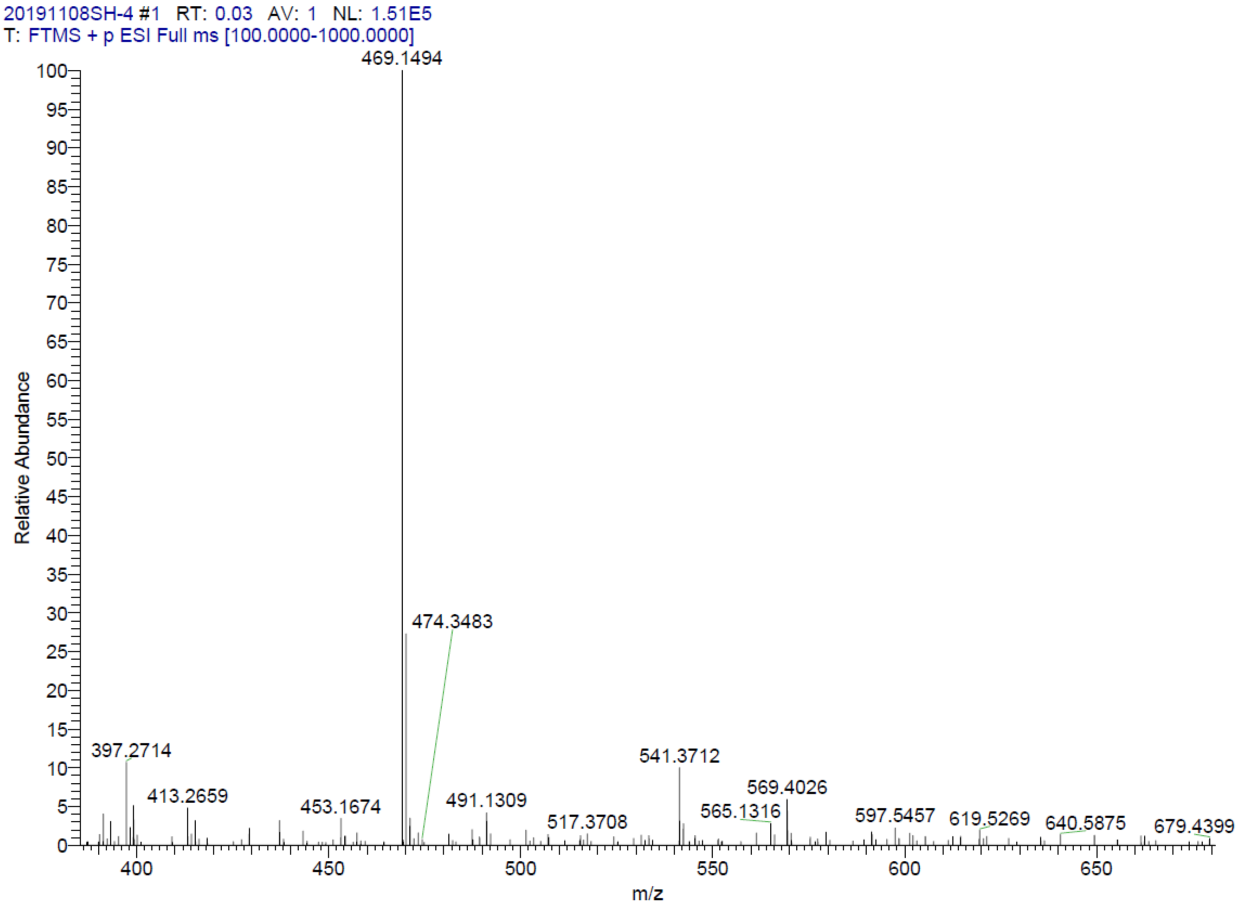

Figure S22. The HR-MS of compound **4**

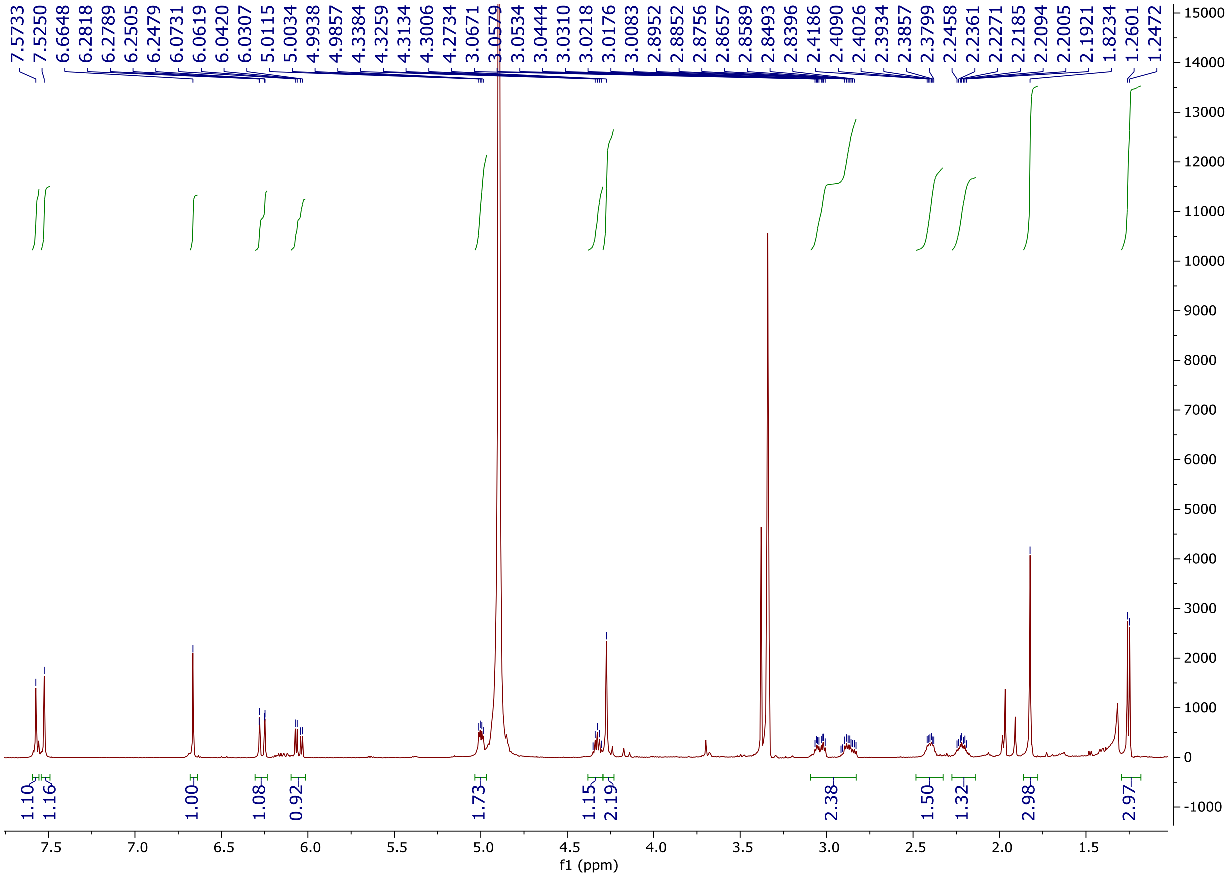

Figure S16. The ^{1}H-NMR of compound **4**

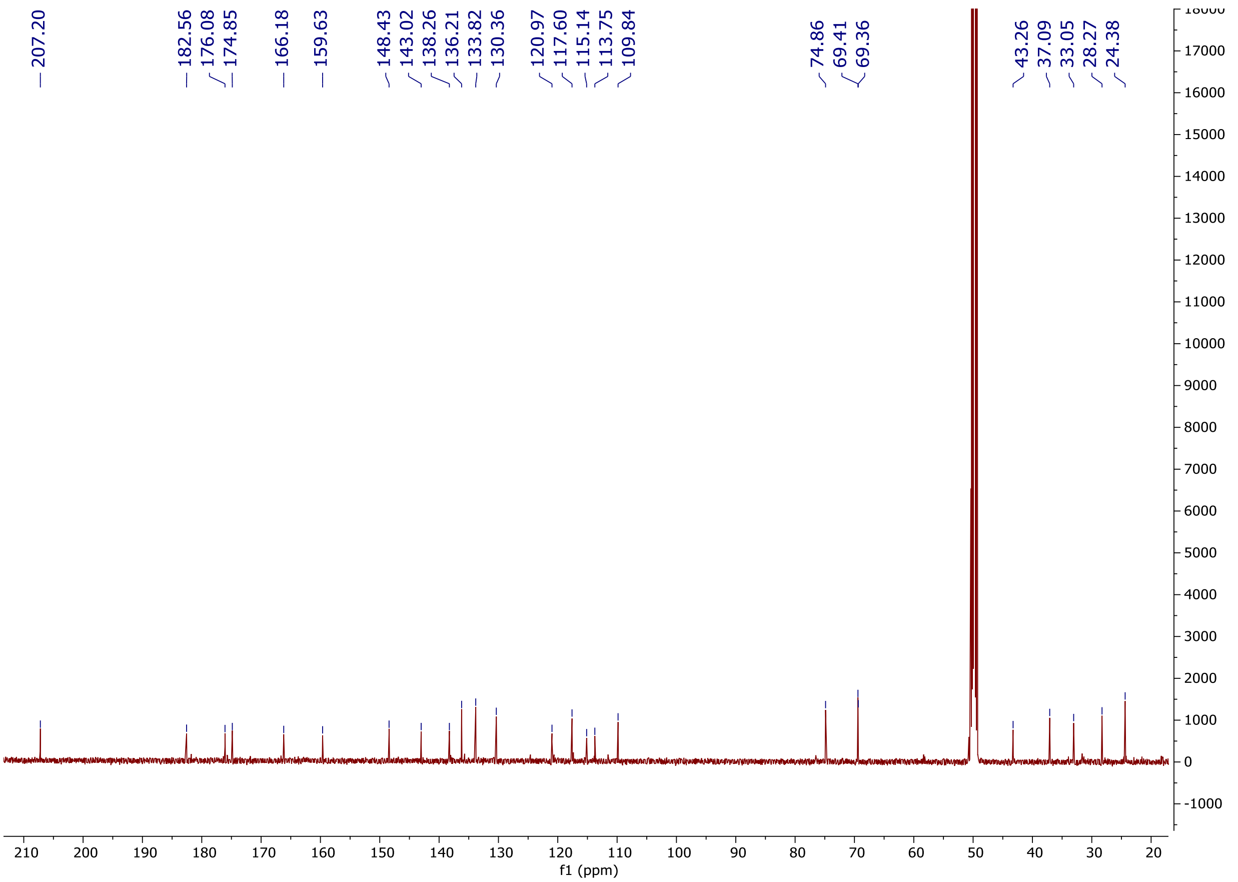

Figure S17. The ^{13}C-NMR of compound **4**

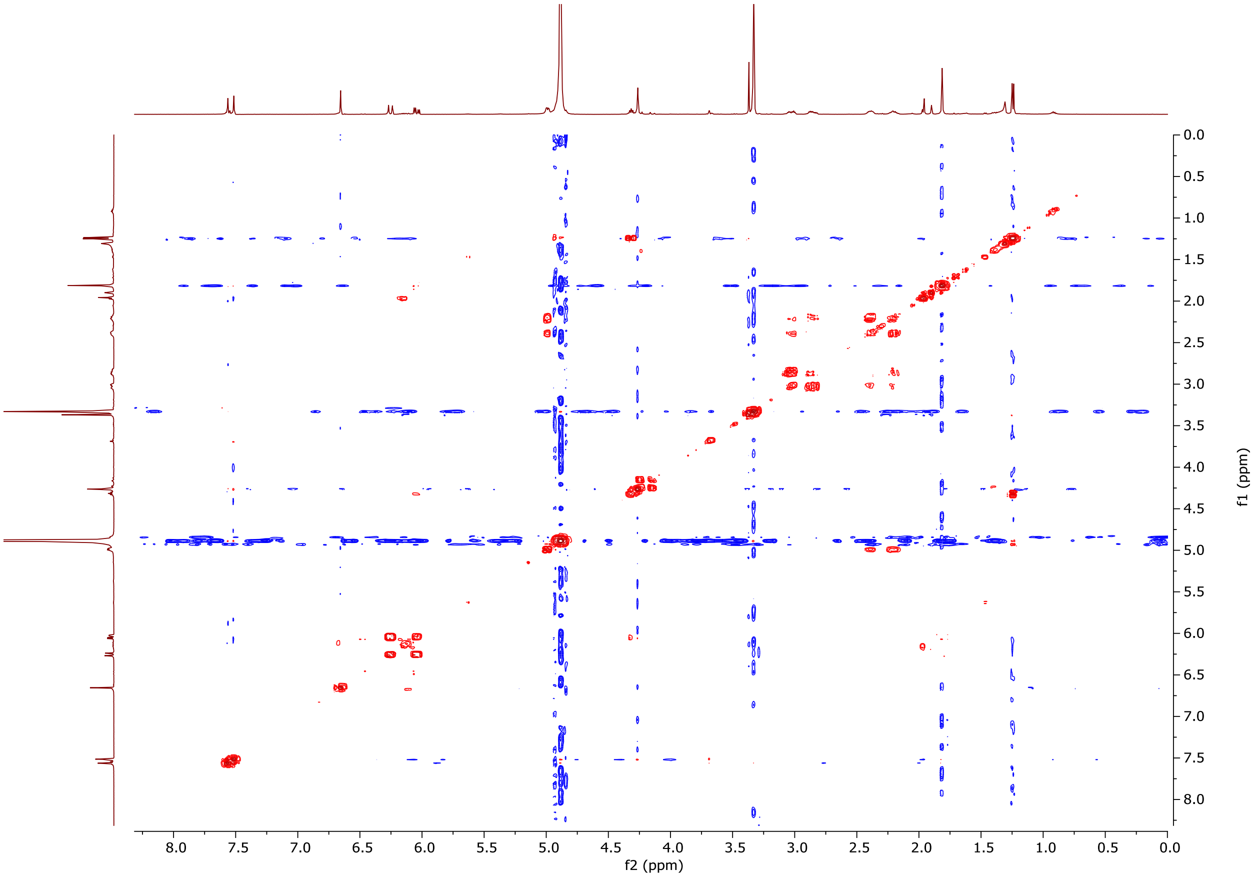

Figure S18. The ^{1}H-^{1}H COSY of compound **4**

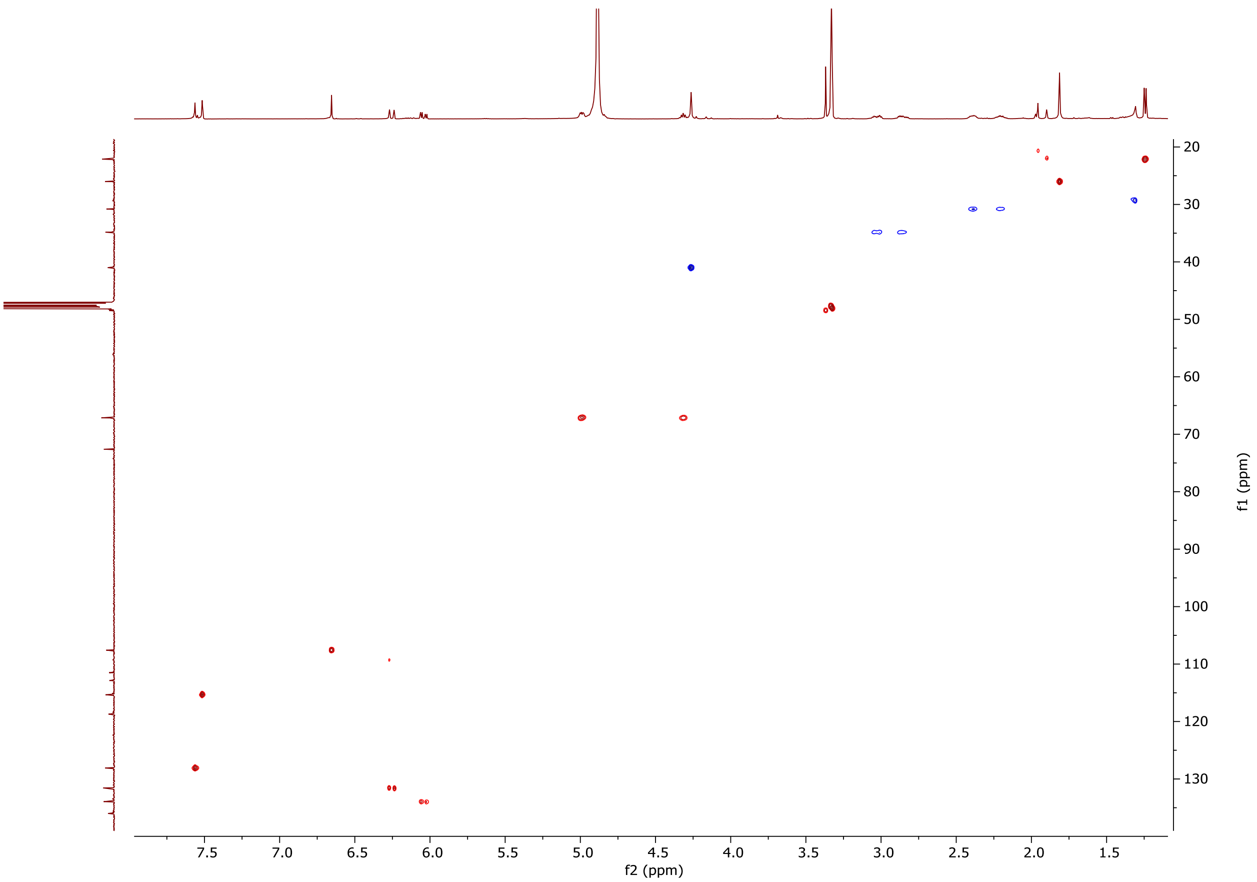

Figure S19. The HSQC of compound **4**

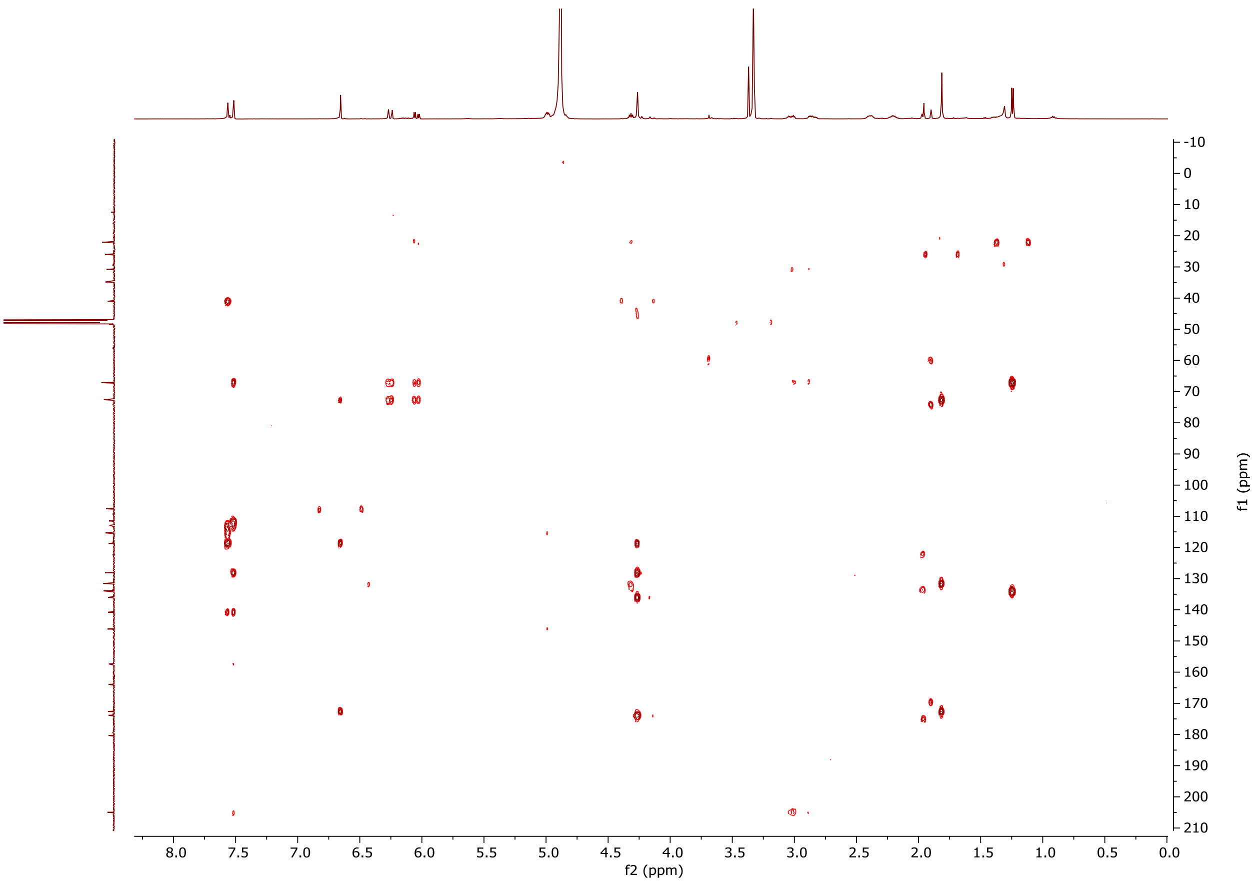

Figure S20. The HMBC of compound **4**

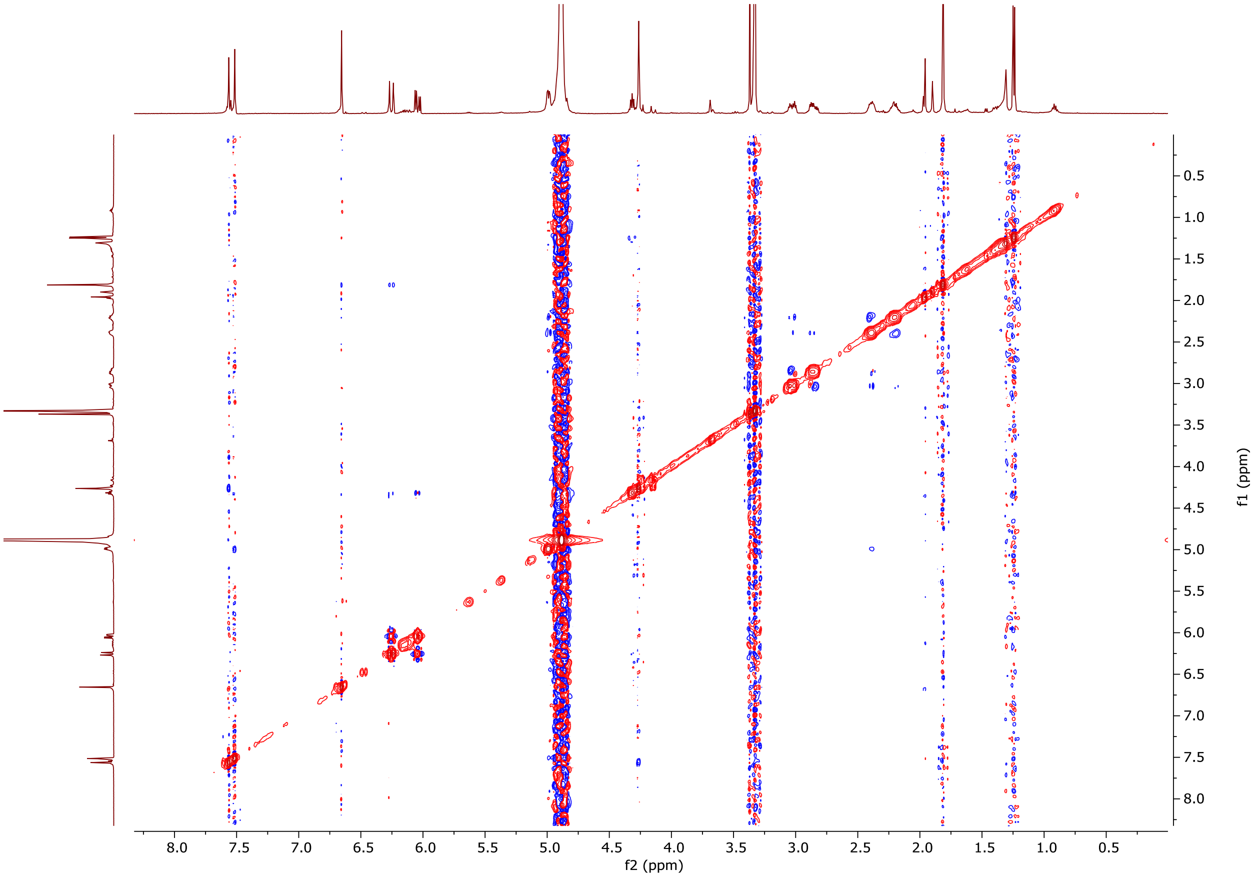

Figure S21. The NOESY of compound **4**

16S ribosomal RNA of *Streptomyces* sp. shell-016, partial sequence

ATGCAGTCGACGATGAACCGGTTTCGGCCGGGGATTAGTGGCGAACGGGTGAGTAA
CACGTGGGCAATCTGCCCTGCACTCTGGGACAAGCCCTGGAAACGGGGTCTAATAC
CGGATATGAACCTGGAAGGCATCTTCCGGGTTGTAAAGCTCCGGCGGTGCAGGATG
AGCCCGCGGCCTATCAGCTTGTTGGTGAGGTAACGGCTCACCAAGGCGACGACGGG
TAGCCGGCCTGAGAGGGCGACCGGCCACACTGGGACTGAGACACGGCCCAGACTCC
TACGGGGAGGCAGCAGTGGGGAATATTGCACAATGGGCGAAAGCCTGATGCAGCGA
CGCCGCGTGAGGGATGACGGCCTTCGGGTTGTAAACCTCTTTCAGCAGGGAAGAAG
CGAAAGTGACGGTACCTGCAGAAGAAGCGCCGGCTAACTACGTGCCAGCAGCCGCG
GTAATACGTAGGGCGCAAGCGTTGTCCGGAATTATTGGGCGTAAAGAGCTCGTAGG
CGGCTTGTCACGTCGGTTGTGAAAGCCCGGGGCTTAACCCCGGGTCTGCAGTCGATA
CGGGCAGGCTAGAGTTCGGTAGGGGAGATCGGAATTCcTGGTGTAGCGGTGAAATG
CGCAGATATCAGGAGGAACACCGGTGGCGAAGGCGGATCTCTGGGCCGATACTGAC
GCTGAGGAGCGAAAGCGTGGGGAGCGAACAGGATTAGATACCCTGGTAGTCCACGC
CGTAAACGGTGGGCACTAGGTGTGGGCAACATTCCACGTTGTCCGTGCCGCAGCTAA
CGCATTAAGTGCCCCGCCTGGGGAGTACGGCCGCAAGGCTAAAACTCAAAGGAATT
GACGGGGGCCCGCACAAGCGGCGGAGCATGTGGCTTAATTCGACGCAACGCGAAGA
ACCTTACCAAGGCTTGACATACACCGGAAAGCATTAGAGATAGTGCCCCCCTTGTGG
TCGGTGTACAGGTGGTGCATGGCTGTCGTCAGCTCGTGTCGTGAGATGTTGGGTTAA
GTCCCGCAACGAGCGCAACCCTTGTCCCGTGTTGCCAGCAGGCCCTTGTGGTGCTGG
GGACTCACGGGAGACCGCCGGGGTCAACTCGGAGGAAGGTGGGGACGACGTCAAG
TCATCATGCCCCTTATGTCTTGGGCTGCACACGTGCTACAATGGCCGGTACAATGAG
CTGCGATACCGCGAGGTGGAGCGAATCTCAAAAAGCCGGTCTCAGTTCGGATTGGG
GTCTGCAACTCGACCCCATGAAGTCGGAGTCGCTAGTAATCGCAGATCAGCATTGCT
GCGGTGAATACGTTCCCGGGCCTTGTACACACCGCCCGTCACGTCACGAAAGTCGGT
AACACCCGAAGCCGGTGGCCCAACCCCTTGTGGGAGGGAGC

Crystal Structure Reports

Experimental Summary

The single crystal X-ray diffraction studies were carried out on a Bruker Kappa Photon II CPAD diffractometer equipped with Cu K_{α} radiation (λ = 1.54178). A 0.217 x 0.114 x 0.096 mm piece of an orange rod was mounted on a Cryoloop with Paratone oil. Data were collected in a nitrogen gas stream at 100(2) K using ϕ and ϖ scans. Crystal-to-detector distance was 50 mm using variable exposure time (10s-60s) depending on θ with a scan width of 1.0°. Data collection was 89.2% complete to 59.031° in θ, (0.90 Å). A total of 14220 reflections were collected covering the indices, -10<=h<=10, -12<=k<=12, -13<=l<=12. 5334 reflections were found to be symmetry independent, with a R_{int} of 0.0251. Indexing and unit cell refinement indicated a primitive, triclinic lattice. The space group was found to be *P*1. The data were integrated using the Bruker SAINT software program and scaled using the SADABS software program. Solution by direct methods (SHELXT) produced a complete phasing model for refinement.

All nonhydrogen atoms were refined anisotropically by full-matrix least-squares (SHELXL-2014). All hydrogen atoms were placed using a riding model. Their positions were constrained relative to their parent atom using the appropriate HFIX command in SHELXL-2014. The absolute stereochemistry of the molecule was established by anomalous dispersion using the Parson's method with a Flack parameter of 0.020(73). Crystallographic data are summarized in Table 1.

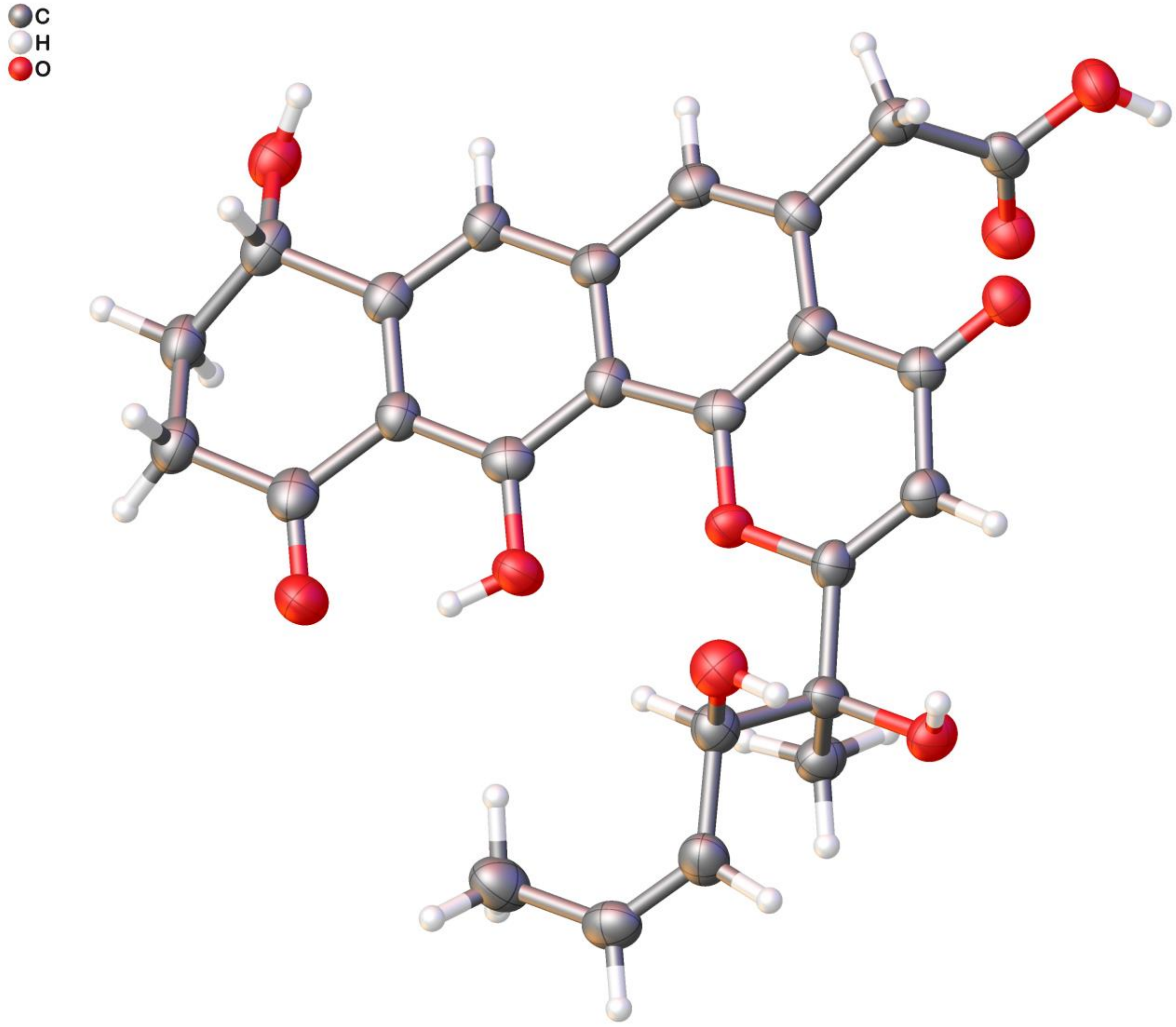
C
H
O

Table 1. Crystal data and structure refinement for SHELLMYCINA.

Report date	2019-12-05	
Identification code	SHELLMYCINA	
Empirical formula	C26.20 H28.40 O10	
Molecular formula	C25 H24 O9, 0.8(C H4 O), 0.2(C2 H6 O)	
Formula weight	503.29	
Temperature	100 K	
Wavelength	1.54178 Å	
Crystal system	Triclinic	
Space group	P1	
Unit cell dimensions	a = 9.5749(5) Å	α= 65.634(3)°.
	b = 11.5552(6) Å	β= 89.720(3)°.
	c = 11.8417(6) Å	γ = 79.122(3)°.
Volume	1168.12(11) $Å^3$	
Z	2	
Density (calculated)	1.431 Mg/m^3	
Absorption coefficient	0.927 mm^{-1}	
F(000)	531	
Crystal size	0.217 x 0.114 x 0.096 mm^3	
Crystal color, habit	Orange Rod	
Theta range for data collection	4.112 to 59.031°.	
Index ranges	-10<=h<=10, -12<=k<=12, -13<=l<=12	
Reflections collected	14220	
Independent reflections	5334 [R(int) = 0.0251, R(sigma) = 0.0313]	
Completeness to theta = 59.031°	89.2 %	
Absorption correction	Semi-empirical from equivalents	
Max. and min. transmission	0.152328 and 0.065793	
Refinement method	Full-matrix least-squares on F^2	
Data / restraints / parameters	5334 / 21 / 676	
Goodness-of-fit on F^2	1.078	
Final R indices [I>2sigma(I)]	R1 = 0.0274, wR2 = 0.0745	
R indices (all data)	R1 = 0.0282, wR2 = 0.0753	
Absolute structure parameter	0.02(7)	
Extinction coefficient	n/a	
Largest diff. peak and hole	0.190 and -0.168 e.$Å^{-3}$	

Table 2. Atomic coordinates (x 10^4) and equivalent isotropic displacement parameters (Å^{2}x 10^3) for SHELLMYCINA. U(eq) is defined as one third of the trace of the orthogonalized U^{ij} tensor.

	x	y	z	U(eq)
O(1)	5155(3)	1211(3)	459(3)	43(1)
O(2)	3957(3)	3950(3)	-229(3)	36(1)
O(3)	3163(3)	1557(3)	2654(3)	30(1)
O(4)	5564(3)	3248(3)	4091(3)	33(1)
O(5)	1705(3)	-132(3)	2856(3)	35(1)
O(6)	598(3)	-2020(3)	3175(3)	44(1)
O(7)	576(3)	-3325(3)	8164(3)	40(1)
O(8)	3095(3)	3812(3)	5609(3)	37(1)
O(9)	5141(3)	3801(3)	6524(3)	38(1)
C(1)	1505(5)	467(5)	-571(5)	48(1)
C(2)	2164(5)	1551(4)	-1385(5)	40(1)
C(3)	3081(5)	2098(4)	-1044(4)	38(1)
C(4)	3656(4)	1747(4)	245(4)	34(1)
C(5)	3291(4)	2938(4)	552(4)	30(1)
C(6)	3751(4)	2570(4)	1898(4)	30(1)
C(7)	4546(4)	3138(4)	2341(4)	32(1)
C(8)	4793(4)	2742(4)	3657(4)	28(1)
C(9)	4077(4)	1709(4)	4464(5)	32(1)
C(10)	3302(4)	1149(4)	3907(4)	30(1)
C(11)	2592(4)	107(4)	4608(5)	30(1)
C(12)	1866(4)	-545(4)	4085(5)	32(1)
C(13)	1362(4)	-1658(4)	4878(5)	30(1)
C(14)	753(4)	-2398(4)	4317(5)	38(1)
C(15)	362(5)	-3645(4)	5155(5)	40(1)
C(16)	-43(4)	-3681(4)	6415(5)	38(1)
C(17)	1083(4)	-3360(4)	7041(5)	35(1)
C(18)	1495(4)	-2077(4)	6183(4)	32(1)
C(19)	2073(4)	-1377(4)	6678(4)	29(1)
C(20)	2668(4)	-316(4)	5914(4)	30(1)
C(21)	3411(4)	318(4)	6458(5)	32(1)
C(22)	4115(4)	1273(4)	5781(4)	29(1)

C(23)	4909(4)	1842(4)	6454(4)	34(1)
C(24)	4268(5)	3257(4)	6122(4)	31(1)
C(25)	1689(4)	3486(4)	349(4)	35(1)
O(1')	8545(3)	5724(2)	9707(3)	32(1)
O(2')	5680(3)	5848(3)	10063(3)	32(1)
O(3')	6643(3)	8192(2)	7226(3)	29(1)
O(4')	4501(3)	6364(3)	5760(3)	34(1)
O(5')	8220(3)	9776(3)	7073(3)	32(1)
O(6')	9556(3)	11522(3)	6795(3)	40(1)
O(7')	8592(3)	13335(3)	1740(3)	36(1)
O(8')	7010(3)	5833(3)	4291(3)	36(1)
O(9')	5030(3)	5842(3)	3279(3)	38(1)
C(1')	8459(6)	9000(5)	10517(5)	48(1)
C(2')	8181(5)	7691(4)	11355(5)	34(1)
C(3')	7862(4)	6809(4)	11024(4)	32(1)
C(4')	7720(4)	6894(4)	9732(4)	32(1)
C(5')	6155(4)	7012(4)	9321(4)	33(1)
C(6')	6009(4)	7210(4)	7974(4)	27(1)
C(7')	5316(4)	6582(4)	7505(4)	30(1)
C(8')	5189(4)	6918(4)	6188(4)	31(1)
C(9')	5909(4)	7953(4)	5407(4)	28(1)
C(10')	6612(4)	8545(4)	5979(4)	27(1)
C(11')	7365(4)	9554(4)	5299(4)	28(1)
C(12')	8137(4)	10172(4)	5844(4)	29(1)
C(13')	8764(4)	11209(4)	5082(5)	29(1)
C(14')	9456(4)	11876(4)	5656(5)	31(1)
C(15')	10047(4)	13039(4)	4824(5)	37(1)
C(16')	9299(5)	13705(4)	3524(4)	35(1)
C(17')	9335(4)	12750(4)	2957(4)	31(1)
C(18')	8679(4)	11621(4)	3770(4)	30(1)
C(19')	8006(5)	10979(4)	3255(4)	33(1)
C(20')	7340(4)	9955(4)	3997(4)	29(1)
C(21')	6599(4)	9355(4)	3431(5)	31(1)
C(22')	5916(4)	8380(4)	4089(4)	30(1)
C(23')	5196(4)	7780(4)	3401(4)	31(1)
C(24')	5858(5)	6381(4)	3729(4)	31(1)

C(25')	5181(5)	8119(4)	9491(4)	35(1)
O(2S)	6938(3)	13273(3)	-115(3)	50(1)
C(2S)	7785(6)	13331(6)	-1134(6)	62(2)
C(4S)	8130(30)	13590(30)	-2480(30)	62(2)
O(1S)	2532(3)	-3496(3)	9875(3)	48(1)
C(1S)	2174(6)	-3475(6)	11081(6)	65(2)
C(3S)	1980(30)	-4030(30)	12370(30)	65(2)

Table 3. Bond lengths [Å] and angles [°] for SHELLMYCINA.

O(1)-H(1)	0.8400	C(11)-C(12)	1.423(7)
O(1)-C(4)	1.430(5)	C(11)-C(20)	1.414(7)
O(2)-H(2)	0.8400	C(12)-C(13)	1.415(6)
O(2)-C(5)	1.418(5)	C(13)-C(14)	1.474(7)
O(3)-C(6)	1.367(5)	C(13)-C(18)	1.414(7)
O(3)-C(10)	1.357(6)	C(14)-C(15)	1.490(6)
O(4)-C(8)	1.251(5)	C(15)-H(15A)	0.9900
O(5)-H(5)	0.8400	C(15)-H(15B)	0.9900
O(5)-C(12)	1.330(6)	C(15)-C(16)	1.526(7)
O(6)-C(14)	1.237(6)	C(16)-H(16A)	0.9900
O(7)-H(7)	0.8400	C(16)-H(16B)	0.9900
O(7)-C(17)	1.426(6)	C(16)-C(17)	1.496(7)
O(8)-C(24)	1.207(5)	C(17)-H(17)	1.0000
O(9)-H(9)	0.8400	C(17)-C(18)	1.536(6)
O(9)-C(24)	1.330(5)	C(18)-C(19)	1.367(6)
C(1)-H(1A)	0.9800	C(19)-H(19)	0.9500
C(1)-H(1B)	0.9800	C(19)-C(20)	1.410(6)
C(1)-H(1C)	0.9800	C(20)-C(21)	1.432(6)
C(1)-C(2)	1.481(7)	C(21)-H(21)	0.9500
C(2)-H(2A)	0.9500	C(21)-C(22)	1.364(6)
C(2)-C(3)	1.326(6)	C(22)-C(23)	1.507(6)
C(3)-H(3)	0.9500	C(23)-H(23A)	0.9900
C(3)-C(4)	1.487(6)	C(23)-H(23B)	0.9900
C(4)-H(4)	1.0000	C(23)-C(24)	1.516(6)
C(4)-C(5)	1.542(6)	C(25)-H(25A)	0.9800
C(5)-C(6)	1.514(6)	C(25)-H(25B)	0.9800
C(5)-C(25)	1.524(6)	C(25)-H(25C)	0.9800
C(6)-C(7)	1.327(6)	O(1')-H(1')	0.8400
C(7)-H(7A)	0.9500	O(1')-C(4')	1.443(5)
C(7)-C(8)	1.439(7)	O(2')-H(2')	0.8400
C(8)-C(9)	1.472(6)	O(2')-C(5')	1.429(5)
C(9)-C(10)	1.394(7)	O(3')-C(6')	1.367(5)
C(9)-C(22)	1.426(7)	O(3')-C(10')	1.359(5)
C(10)-C(11)	1.442(6)	O(4')-C(8')	1.238(5)

O(5')-H(5')	0.8400	C(16')-H(16C)	0.9900
O(5')-C(12')	1.331(6)	C(16')-H(16D)	0.9900
O(6')-C(14')	1.236(6)	C(16')-C(17')	1.506(7)
O(7')-H(7')	0.8400	C(17')-H(17')	1.0000
O(7')-C(17')	1.437(5)	C(17')-C(18')	1.513(6)
O(8')-C(24')	1.207(5)	C(18')-C(19')	1.377(7)
O(9')-H(9')	0.8400	C(19')-H(19')	0.9500
O(9')-C(24')	1.328(5)	C(19')-C(20')	1.414(6)
C(1')-H(1'A)	0.9800	C(20')-C(21')	1.417(6)
C(1')-H(1'B)	0.9800	C(21')-H(21')	0.9500
C(1')-H(1'C)	0.9800	C(21')-C(22')	1.361(6)
C(1')-C(2')	1.501(7)	C(22')-C(23')	1.507(6)
C(2')-H(2'A)	0.9500	C(23')-H(23C)	0.9900
C(2')-C(3')	1.317(6)	C(23')-H(23D)	0.9900
C(3')-H(3')	0.9500	C(23')-C(24')	1.505(6)
C(3')-C(4')	1.497(6)	C(25')-H(25D)	0.9800
C(4')-H(4')	1.0000	C(25')-H(25E)	0.9800
C(4')-C(5')	1.542(6)	C(25')-H(25F)	0.9800
C(5')-C(6')	1.518(7)	O(2S)-H(2S)	0.8400
C(5')-C(25')	1.519(6)	O(2S)-C(2S)	1.436(7)
C(6')-C(7')	1.339(6)	C(2S)-H(2SA)	0.9800
C(7')-H(7'A)	0.9500	C(2S)-H(2SB)	0.9800
C(7')-C(8')	1.443(7)	C(2S)-H(2SC)	0.9800
C(8')-C(9')	1.467(6)	C(2S)-H(2SD)	0.9900
C(9')-C(10')	1.393(6)	C(2S)-H(2SE)	0.9900
C(9')-C(22')	1.430(7)	C(2S)-C(4S)	1.54(3)
C(10')-C(11')	1.436(6)	C(4S)-H(4SA)	0.9800
C(11')-C(12')	1.434(7)	C(4S)-H(4SB)	0.9800
C(11')-C(20')	1.414(7)	C(4S)-H(4SC)	0.9800
C(12')-C(13')	1.408(6)	O(1S)-H(1S)	0.8400
C(13')-C(14')	1.458(7)	O(1S)-C(1S)	1.475(8)
C(13')-C(18')	1.423(7)	C(1S)-H(1SA)	0.9800
C(14')-C(15')	1.512(6)	C(1S)-H(1SB)	0.9800
C(15')-H(15C)	0.9900	C(1S)-H(1SC)	0.9800
C(15')-H(15D)	0.9900	C(1S)-H(1SD)	0.9900
C(15')-C(16')	1.517(6)	C(1S)-H(1SE)	0.9900

C(1S)-C(3S)	1.42(3)	C(7)-C(6)-O(3)	122.5(4)
C(3S)-H(3SA)	0.9800	C(7)-C(6)-C(5)	127.8(4)
C(3S)-H(3SB)	0.9800	C(6)-C(7)-H(7A)	119.4
C(3S)-H(3SC)	0.9800	C(6)-C(7)-C(8)	121.2(4)
		C(8)-C(7)-H(7A)	119.4
C(4)-O(1)-H(1)	109.5	O(4)-C(8)-C(7)	122.0(4)
C(5)-O(2)-H(2)	109.5	O(4)-C(8)-C(9)	122.0(4)
C(10)-O(3)-C(6)	120.2(4)	C(7)-C(8)-C(9)	116.0(4)
C(12)-O(5)-H(5)	109.5	C(10)-C(9)-C(8)	118.2(4)
C(17)-O(7)-H(7)	109.5	C(10)-C(9)-C(22)	118.5(4)
C(24)-O(9)-H(9)	109.5	C(22)-C(9)-C(8)	123.3(4)
H(1A)-C(1)-H(1B)	109.5	O(3)-C(10)-C(9)	121.8(4)
H(1A)-C(1)-H(1C)	109.5	O(3)-C(10)-C(11)	115.3(4)
H(1B)-C(1)-H(1C)	109.5	C(9)-C(10)-C(11)	122.9(5)
C(2)-C(1)-H(1A)	109.5	C(12)-C(11)-C(10)	124.7(5)
C(2)-C(1)-H(1B)	109.5	C(20)-C(11)-C(10)	117.1(4)
C(2)-C(1)-H(1C)	109.5	C(20)-C(11)-C(12)	118.1(4)
C(1)-C(2)-H(2A)	116.3	O(5)-C(12)-C(11)	119.9(4)
C(3)-C(2)-C(1)	127.4(5)	O(5)-C(12)-C(13)	120.4(4)
C(3)-C(2)-H(2A)	116.3	C(13)-C(12)-C(11)	119.6(5)
C(2)-C(3)-H(3)	116.6	C(12)-C(13)-C(14)	118.5(5)
C(2)-C(3)-C(4)	126.8(4)	C(18)-C(13)-C(12)	120.5(4)
C(4)-C(3)-H(3)	116.6	C(18)-C(13)-C(14)	121.0(4)
O(1)-C(4)-C(3)	113.1(4)	O(6)-C(14)-C(13)	121.3(4)
O(1)-C(4)-H(4)	107.5	O(6)-C(14)-C(15)	120.0(5)
O(1)-C(4)-C(5)	111.1(3)	C(13)-C(14)-C(15)	118.6(5)
C(3)-C(4)-H(4)	107.5	C(14)-C(15)-H(15A)	109.0
C(3)-C(4)-C(5)	109.9(3)	C(14)-C(15)-H(15B)	109.0
C(5)-C(4)-H(4)	107.5	C(14)-C(15)-C(16)	112.9(4)
O(2)-C(5)-C(4)	111.2(3)	H(15A)-C(15)-H(15B)	107.8
O(2)-C(5)-C(6)	109.3(3)	C(16)-C(15)-H(15A)	109.0
O(2)-C(5)-C(25)	106.9(3)	C(16)-C(15)-H(15B)	109.0
C(6)-C(5)-C(4)	110.6(3)	C(15)-C(16)-H(16A)	109.2
C(6)-C(5)-C(25)	107.9(4)	C(15)-C(16)-H(16B)	109.2
C(25)-C(5)-C(4)	110.8(3)	H(16A)-C(16)-H(16B)	107.9
O(3)-C(6)-C(5)	109.6(3)	C(17)-C(16)-C(15)	112.1(4)

C(17)-C(16)-H(16A)	109.2	H(25A)-C(25)-H(25C)	109.5
C(17)-C(16)-H(16B)	109.2	H(25B)-C(25)-H(25C)	109.5
O(7)-C(17)-C(16)	108.4(3)	C(4')-O(1')-H(1')	109.5
O(7)-C(17)-H(17)	108.3	C(5')-O(2')-H(2')	109.5
O(7)-C(17)-C(18)	111.8(4)	C(10')-O(3')-C(6')	120.7(3)
C(16)-C(17)-H(17)	108.3	C(12')-O(5')-H(5')	109.5
C(16)-C(17)-C(18)	111.6(4)	C(17')-O(7')-H(7')	109.5
C(18)-C(17)-H(17)	108.3	C(24')-O(9')-H(9')	109.5
C(13)-C(18)-C(17)	120.0(4)	H(1'A)-C(1')-H(1'B)	109.5
C(19)-C(18)-C(13)	119.7(4)	H(1'A)-C(1')-H(1'C)	109.5
C(19)-C(18)-C(17)	120.2(4)	H(1'B)-C(1')-H(1'C)	109.5
C(18)-C(19)-H(19)	119.6	C(2')-C(1')-H(1'A)	109.5
C(18)-C(19)-C(20)	120.7(4)	C(2')-C(1')-H(1'B)	109.5
C(20)-C(19)-H(19)	119.6	C(2')-C(1')-H(1'C)	109.5
C(11)-C(20)-C(21)	118.8(4)	C(1')-C(2')-H(2'A)	116.4
C(19)-C(20)-C(11)	120.9(4)	C(3')-C(2')-C(1')	127.3(5)
C(19)-C(20)-C(21)	120.2(5)	C(3')-C(2')-H(2'A)	116.4
C(20)-C(21)-H(21)	118.5	C(2')-C(3')-H(3')	116.4
C(22)-C(21)-C(20)	123.0(5)	C(2')-C(3')-C(4')	127.2(4)
C(22)-C(21)-H(21)	118.5	C(4')-C(3')-H(3')	116.4
C(9)-C(22)-C(23)	121.9(4)	O(1')-C(4')-C(3')	108.9(3)
C(21)-C(22)-C(9)	119.4(4)	O(1')-C(4')-H(4')	109.6
C(21)-C(22)-C(23)	118.7(4)	O(1')-C(4')-C(5')	107.9(3)
C(22)-C(23)-H(23A)	109.1	C(3')-C(4')-H(4')	109.6
C(22)-C(23)-H(23B)	109.1	C(3')-C(4')-C(5')	111.2(3)
C(22)-C(23)-C(24)	112.7(4)	C(5')-C(4')-H(4')	109.6
H(23A)-C(23)-H(23B)	107.8	O(2')-C(5')-C(4')	110.0(3)
C(24)-C(23)-H(23A)	109.1	O(2')-C(5')-C(6')	107.7(3)
C(24)-C(23)-H(23B)	109.1	O(2')-C(5')-C(25')	107.3(3)
O(8)-C(24)-O(9)	123.8(4)	C(6')-C(5')-C(4')	110.7(3)
O(8)-C(24)-C(23)	124.9(4)	C(6')-C(5')-C(25')	110.0(3)
O(9)-C(24)-C(23)	111.2(4)	C(25')-C(5')-C(4')	111.1(4)
C(5)-C(25)-H(25A)	109.5	O(3')-C(6')-C(5')	111.1(4)
C(5)-C(25)-H(25B)	109.5	C(7')-C(6')-O(3')	121.4(4)
C(5)-C(25)-H(25C)	109.5	C(7')-C(6')-C(5')	127.4(4)
H(25A)-C(25)-H(25B)	109.5	C(6')-C(7')-H(7'A)	119.1

C(6')-C(7')-C(8')	121.7(4)	O(7')-C(17')-H(17')	108.3
C(8')-C(7')-H(7'A)	119.1	O(7')-C(17')-C(18')	108.3(3)
O(4')-C(8')-C(7')	121.2(4)	C(16')-C(17')-H(17')	108.3
O(4')-C(8')-C(9')	122.9(4)	C(16')-C(17')-C(18')	111.1(4)
C(7')-C(8')-C(9')	115.9(4)	C(18')-C(17')-H(17')	108.3
C(10')-C(9')-C(8')	118.3(4)	C(13')-C(18')-C(17')	119.9(4)
C(10')-C(9')-C(22')	118.8(4)	C(19')-C(18')-C(13')	119.4(4)
C(22')-C(9')-C(8')	122.9(4)	C(19')-C(18')-C(17')	120.7(4)
O(3')-C(10')-C(9')	121.9(3)	C(18')-C(19')-H(19')	119.3
O(3')-C(10')-C(11')	115.5(4)	C(18')-C(19')-C(20')	121.3(5)
C(9')-C(10')-C(11')	122.6(4)	C(20')-C(19')-H(19')	119.3
C(12')-C(11')-C(10')	124.9(4)	C(11')-C(20')-C(19')	120.5(4)
C(20')-C(11')-C(10')	117.0(4)	C(11')-C(20')-C(21')	119.5(4)
C(20')-C(11')-C(12')	118.1(4)	C(19')-C(20')-C(21')	119.9(4)
O(5')-C(12')-C(11')	119.1(4)	C(20')-C(21')-H(21')	118.5
O(5')-C(12')-C(13')	120.6(4)	C(22')-C(21')-C(20')	122.9(5)
C(13')-C(12')-C(11')	120.3(5)	C(22')-C(21')-H(21')	118.5
C(12')-C(13')-C(14')	118.9(5)	C(9')-C(22')-C(23')	122.1(4)
C(12')-C(13')-C(18')	120.3(4)	C(21')-C(22')-C(9')	119.2(4)
C(18')-C(13')-C(14')	120.8(4)	C(21')-C(22')-C(23')	118.7(4)
O(6')-C(14')-C(13')	121.7(4)	C(22')-C(23')-H(23C)	108.7
O(6')-C(14')-C(15')	119.7(4)	C(22')-C(23')-H(23D)	108.7
C(13')-C(14')-C(15')	118.6(4)	H(23C)-C(23')-H(23D)	107.6
C(14')-C(15')-H(15C)	109.1	C(24')-C(23')-C(22')	114.0(4)
C(14')-C(15')-H(15D)	109.1	C(24')-C(23')-H(23C)	108.7
C(14')-C(15')-C(16')	112.5(4)	C(24')-C(23')-H(23D)	108.7
H(15C)-C(15')-H(15D)	107.8	O(8')-C(24')-O(9')	124.4(4)
C(16')-C(15')-H(15C)	109.1	O(8')-C(24')-C(23')	124.5(4)
C(16')-C(15')-H(15D)	109.1	O(9')-C(24')-C(23')	111.1(3)
C(15')-C(16')-H(16C)	109.5	C(5')-C(25')-H(25D)	109.5
C(15')-C(16')-H(16D)	109.5	C(5')-C(25')-H(25E)	109.5
H(16C)-C(16')-H(16D)	108.1	C(5')-C(25')-H(25F)	109.5
C(17')-C(16')-C(15')	110.6(3)	H(25D)-C(25')-H(25E)	109.5
C(17')-C(16')-H(16C)	109.5	H(25D)-C(25')-H(25F)	109.5
C(17')-C(16')-H(16D)	109.5	H(25E)-C(25')-H(25F)	109.5
O(7')-C(17')-C(16')	112.3(3)	C(2S)-O(2S)-H(2S)	109.5

O(2S)-C(2S)-H(2SA)	109.5	H(3SB)-C(3S)-H(3SC)	109.5
O(2S)-C(2S)-H(2SB)	109.5		
O(2S)-C(2S)-H(2SC)	109.5		
O(2S)-C(2S)-H(2SD)	96.7		
O(2S)-C(2S)-H(2SE)	96.7		
O(2S)-C(2S)-C(4S)	158.4(12)		
H(2SA)-C(2S)-H(2SB)	109.5		
H(2SA)-C(2S)-H(2SC)	109.5		
H(2SB)-C(2S)-H(2SC)	109.5		
H(2SD)-C(2S)-H(2SE)	103.5		
C(4S)-C(2S)-H(2SD)	96.7		
C(4S)-C(2S)-H(2SE)	96.7		
C(2S)-C(4S)-H(4SA)	109.5		
C(2S)-C(4S)-H(4SB)	109.5		
C(2S)-C(4S)-H(4SC)	109.5		
H(4SA)-C(4S)-H(4SB)	109.5		
H(4SA)-C(4S)-H(4SC)	109.5		
H(4SB)-C(4S)-H(4SC)	109.5		
C(1S)-O(1S)-H(1S)	109.5		
O(1S)-C(1S)-H(1SA)	109.5		
O(1S)-C(1S)-H(1SB)	109.5		
O(1S)-C(1S)-H(1SC)	109.5		
O(1S)-C(1S)-H(1SD)	97.5		
O(1S)-C(1S)-H(1SE)	97.5		
H(1SA)-C(1S)-H(1SB)	109.5		
H(1SA)-C(1S)-H(1SC)	109.5		
H(1SB)-C(1S)-H(1SC)	109.5		
H(1SD)-C(1S)-H(1SE)	103.6		
C(3S)-C(1S)-O(1S)	155.5(14)		
C(3S)-C(1S)-H(1SD)	97.5		
C(3S)-C(1S)-H(1SE)	97.5		
C(1S)-C(3S)-H(3SA)	109.5		
C(1S)-C(3S)-H(3SB)	109.5		
C(1S)-C(3S)-H(3SC)	109.5		
H(3SA)-C(3S)-H(3SB)	109.5		
H(3SA)-C(3S)-H(3SC)	109.5		

Table 4. Anisotropic displacement parameters ($Å^2 x 10^3$) for SHELLMYCINA. The anisotropic displacement factor exponent takes the form: $-2\pi^2[h^2 a^{*2}U^{11} + ... + 2 h k a^* b^* U^{12}]$

	U^{11}	U^{22}	U^{33}	U^{23}	U^{13}	U^{12}
O(1)	40(2)	43(2)	45(2)	-19(2)	5(2)	-6(1)
O(2)	35(2)	37(2)	32(2)	-9(1)	5(1)	-13(1)
O(3)	32(2)	34(2)	24(2)	-11(1)	5(1)	-10(1)
O(4)	30(2)	36(2)	36(2)	-18(1)	4(1)	-11(1)
O(5)	38(2)	41(2)	31(2)	-17(2)	2(1)	-13(1)
O(6)	49(2)	46(2)	39(3)	-16(2)	-3(2)	-17(1)
O(7)	38(2)	38(2)	40(2)	-12(2)	7(2)	-10(1)
O(8)	32(2)	39(2)	43(2)	-19(1)	2(2)	-6(1)
O(9)	39(2)	35(2)	44(2)	-20(2)	-1(2)	-10(1)
C(1)	45(3)	61(3)	44(4)	-24(3)	4(2)	-20(2)
C(2)	44(3)	45(2)	32(3)	-19(2)	2(2)	-3(2)
C(3)	46(3)	39(2)	34(3)	-19(2)	9(2)	-13(2)
C(4)	32(2)	36(2)	35(3)	-15(2)	6(2)	-10(2)
C(5)	32(2)	34(2)	25(3)	-11(2)	2(2)	-12(2)
C(6)	29(2)	29(2)	32(3)	-11(2)	7(2)	-9(2)
C(7)	29(2)	35(2)	31(3)	-12(2)	5(2)	-9(2)
C(8)	26(2)	31(2)	29(3)	-13(2)	4(2)	-4(2)
C(9)	29(2)	29(2)	33(3)	-11(2)	2(2)	-2(2)
C(10)	26(2)	31(2)	31(3)	-14(2)	0(2)	2(2)
C(11)	24(2)	28(2)	35(3)	-11(2)	3(2)	-4(2)
C(12)	25(2)	35(2)	33(3)	-14(2)	1(2)	-1(2)
C(13)	27(2)	28(2)	35(3)	-12(2)	3(2)	-6(2)
C(14)	29(2)	34(2)	45(4)	-13(2)	-2(2)	-3(2)
C(15)	40(2)	30(2)	44(3)	-10(2)	2(2)	-7(2)
C(16)	33(2)	29(2)	47(3)	-9(2)	2(2)	-9(2)
C(17)	28(2)	31(2)	40(3)	-9(2)	8(2)	-4(2)
C(18)	24(2)	31(2)	36(3)	-11(2)	4(2)	-3(2)
C(19)	26(2)	29(2)	31(3)	-11(2)	5(2)	-5(2)
C(20)	27(2)	29(2)	29(3)	-10(2)	-1(2)	-1(2)
C(21)	29(2)	31(2)	29(3)	-9(2)	-1(2)	-2(2)
C(22)	26(2)	31(2)	32(3)	-17(2)	2(2)	-4(2)

C(23)	34(2)	34(2)	30(3)	-11(2)	4(2)	-7(2)
C(24)	35(3)	38(2)	27(3)	-18(2)	9(2)	-13(2)
C(25)	36(2)	36(2)	30(3)	-10(2)	3(2)	-10(2)
O(1')	28(1)	33(1)	33(2)	-12(1)	6(1)	-7(1)
O(2')	32(2)	29(1)	32(2)	-10(1)	4(1)	-8(1)
O(3')	28(1)	31(1)	29(2)	-12(1)	4(1)	-9(1)
O(4')	33(2)	39(2)	34(2)	-18(1)	5(1)	-11(1)
O(5')	38(2)	34(2)	28(2)	-14(1)	2(1)	-15(1)
O(6')	49(2)	42(2)	30(2)	-13(2)	2(2)	-19(1)
O(7')	37(2)	33(2)	32(2)	-7(1)	4(1)	-10(1)
O(8')	34(2)	36(2)	41(2)	-20(1)	1(2)	-6(1)
O(9')	42(2)	36(1)	42(2)	-20(1)	0(1)	-12(1)
C(1')	60(3)	54(3)	40(3)	-25(2)	9(2)	-23(2)
C(2')	33(2)	44(2)	27(2)	-16(2)	0(2)	-9(2)
C(3')	28(2)	38(2)	26(2)	-9(2)	3(2)	-7(2)
C(4')	35(2)	30(2)	31(2)	-12(2)	5(2)	-8(2)
C(5')	31(2)	28(2)	37(3)	-8(2)	4(2)	-9(2)
C(6')	26(2)	26(2)	28(3)	-8(2)	4(2)	-6(2)
C(7')	26(2)	31(2)	30(3)	-10(2)	3(2)	-7(2)
C(8')	25(2)	31(2)	37(3)	-15(2)	4(2)	-5(2)
C(9')	24(2)	28(2)	30(3)	-11(2)	1(2)	1(2)
C(10')	23(2)	31(2)	28(3)	-13(2)	5(2)	-6(2)
C(11')	26(2)	28(2)	29(3)	-13(2)	2(2)	-1(2)
C(12')	29(2)	29(2)	27(3)	-11(2)	2(2)	-4(2)
C(13')	23(2)	31(2)	35(3)	-15(2)	2(2)	-4(2)
C(14')	28(2)	34(2)	32(3)	-15(2)	2(2)	-6(2)
C(15')	36(2)	33(2)	43(3)	-16(2)	2(2)	-10(2)
C(16')	32(2)	30(2)	38(3)	-9(2)	4(2)	-8(2)
C(17')	30(2)	32(2)	29(3)	-10(2)	4(2)	-7(2)
C(18')	28(2)	31(2)	31(3)	-13(2)	7(2)	-5(2)
C(19')	33(2)	33(2)	28(3)	-11(2)	4(2)	-2(2)
C(20')	26(2)	29(2)	30(3)	-13(2)	5(2)	-2(2)
C(21')	31(2)	36(2)	26(3)	-15(2)	2(2)	-3(2)
C(22')	25(2)	30(2)	34(3)	-14(2)	3(2)	0(2)
C(23')	30(2)	37(2)	30(3)	-17(2)	2(2)	-12(2)
C(24')	33(2)	33(2)	29(3)	-12(2)	7(2)	-11(2)

C(25')	33(2)	33(2)	35(3)	-12(2)	5(2)	-3(2)
O(2S)	40(2)	62(2)	43(2)	-19(2)	2(2)	-11(2)
C(2S)	49(3)	74(4)	73(4)	-41(3)	9(3)	-10(3)
C(4S)	49(3)	74(4)	73(4)	-41(3)	9(3)	-10(3)
O(1S)	37(2)	48(2)	58(2)	-21(2)	9(2)	-9(1)
C(1S)	54(3)	67(3)	68(3)	-24(3)	13(3)	-8(3)
C(3S)	54(3)	67(3)	68(3)	-24(3)	13(3)	-8(3)

Table 5. Hydrogen coordinates (x 10^4) and isotropic displacement parameters ($Å^2$x 10^3) for SHELLMYCINA.

	x	y	z	U(eq)
H(1)	5593	1743	-49	64
H(2)	4845	3679	-142	54
H(5)	1396	-679	2685	53
H(7)	1272	-3426	8648	60
H(9)	4841	4609	6214	57
H(1A)	1978	85	271	72
H(1B)	1614	-195	-898	72
H(1C)	489	794	-548	72
H(2A)	1899	1894	-2252	48
H(3)	3404	2787	-1691	46
H(4)	3161	1070	823	41
H(7A)	4959	3819	1778	38
H(15A)	1179	-4368	5287	48
H(15B)	-451	-3781	4744	48
H(16A)	-950	-3053	6298	46
H(16B)	-200	-4558	6957	46
H(17)	1951	-4070	7262	42
H(19)	2075	-1608	7547	35
H(21)	3414	62	7330	38
H(23A)	5915	1762	6245	41
H(23B)	4903	1337	7362	41
H(25A)	1469	4177	637	53
H(25B)	1183	2793	817	53
H(25C)	1385	3838	-539	53
H(1')	9196	5910	9226	48
H(2')	6216	5213	10005	48
H(5')	8681	10227	7266	48
H(7')	8910	13992	1289	54
H(9')	5358	5039	3558	57
H(1'A)	7606	9668	10410	72
H(1'B)	8689	9012	9705	72

H(1'C)	9262	9173	10888	72
H(2'A)	8241	7475	12222	41
H(3')	7705	6033	11677	38
H(4')	8085	7662	9142	38
H(7'A)	4898	5897	8054	36
H(15C)	11077	12755	4770	44
H(15D)	9944	13669	5202	44
H(16C)	8295	14095	3561	42
H(16D)	9774	14411	2997	42
H(17')	10354	12405	2883	38
H(19')	7989	11229	2383	39
H(21')	6579	9645	2554	37
H(23C)	4181	7844	3582	37
H(23D)	5226	8287	2497	37
H(25D)	4195	8173	9223	53
H(25E)	5475	8937	8990	53
H(25F)	5244	7961	10370	53
H(2S)	7460	13214	482	75
H(2SA)	7222	13233	-1765	93
H(2SB)	8635	12631	-831	93
H(2SC)	8077	14170	-1500	93
H(2SD)	8326	12423	-739	74
H(2SE)	8447	13831	-995	74
H(4SA)	7970	12863	-2658	93
H(4SB)	9128	13665	-2578	93
H(4SC)	7507	14393	-3059	93
H(1S)	3406	-3808	9912	72
H(1SA)	2931	-3201	11403	98
H(1SB)	2087	-4346	11672	98
H(1SC)	1268	-2865	10968	98
H(1SD)	2814	-2901	11099	78
H(1SE)	1228	-2885	10797	78
H(3SA)	1428	-4707	12552	98
H(3SB)	1468	-3352	12605	98
H(3SC)	2915	-4405	12845	98

Table 6. Hydrogen bonds for SHELLMYCINA [Å and °].

D-H...A	d(D-H)	d(H...A)	d(D...A)	<(DHA)
O(1)-H(1)...O(2S)#1	0.84	2.35	3.040(4)	139.4
O(2)-H(2)...O(2S)#1	0.84	1.97	2.802(4)	172.4
O(5)-H(5)...O(6)	0.84	1.74	2.492(4)	148.4
O(7)-H(7)...O(1S)	0.84	1.86	2.687(5)	168.2
O(9)-H(9)...O(4')	0.84	1.83	2.659(4)	167.6
O(1')-H(1')...O(7)#2	0.84	1.90	2.729(4)	167.8
O(2')-H(2')...O(2S)#3	0.84	2.28	3.084(5)	160.2
O(5')-H(5')...O(6')	0.84	1.74	2.497(4)	148.8
O(7')-H(7')...O(1')#4	0.84	2.07	2.809(4)	146.4
O(9')-H(9')...O(4)	0.84	1.87	2.687(4)	164.2
O(2S)-H(2S)...O(7')	0.84	1.91	2.747(5)	172.1
O(1S)-H(1S)...O(2')#1	0.84	2.13	2.952(4)	164.8

Symmetry transformations used to generate equivalent atoms:

#1 x,y-1,z #2 x+1,y+1,z #3 x,y-1,z+1 #4 x,y+1,z-1
